# Comparative Antagonistic Activities of Endolichenic Fungi Isolated from the Fruticose Lichens *Ramalina* and *Usnea*

**DOI:** 10.3390/jof11040302

**Published:** 2025-04-10

**Authors:** Lloyd Christian Jamilano-Llames, Thomas Edison E. dela Cruz

**Affiliations:** 1 The Graduate School, University of Santo Tomas, España Blvd., Manila 1015, Philippines; lloydchristian.llames.gs@ust.edu.ph; 2 Fungal Biodiversity, Ecogenomics and Systematics-Metabolomics (FBeS) Group, Research Center for the Natural and Applied Sciences (RCNAS), University of Santo Tomas, España Blvd., Manila 1015, Philippines; 3 Department of Biological Sciences, College of Science, University of Santo Tomas, España Blvd., Manila 1015, Philippines

**Keywords:** antifungal activity, fungal pathogen, lichen symbiosis, lichen-associated fungi, thalli

## Abstract

Persistent fungal pathogens remain a threat to global food security as these pathogens continue to infect crops despite different mitigating strategies. Traditionally, synthetic fungicides are used to combat these threats, but their environmental and health impacts have spurred interest in a more sustainable, eco-friendly approach. Endolichenic fungi (ELF) are a relatively underexplored group of microorganisms found thriving inside the lichen thalli. They are seen as promising alternatives for developing sustainable plant disease management strategies. Hence, in this study, a total of forty ELF isolates from two fruticose lichen hosts—*Ramalina* and *Usnea*, were tested and compared for their antagonistic activities against three economically important filamentous fungal pathogens—*Colletotrichum gloeosporioides*, *Cladosporium cladosporioides*, and *Fusarium oxysporum*. The results of the dual culture assay showed that all ELF isolates successfully reduced the growth of the three filamentous fungal pathogens with varying degrees, and with direct contact inhibition as the predominant trait among the endolichenic fungi. Comparing the antagonistic activities between the different endolichenic fungi from the two lichen hosts, ELF isolates from *Ramalina* generally demonstrated a higher percentage inhibition of growth of the test fungi as compared to ELF isolates from *Usnea*. This study underscores the importance of endolichenic fungi as an efficient biocontrol agent.

## 1. Introduction

Plant fungal pathogens have been a persistent problem for global food security, causing extensive crop damage and notable economic losses annually [1]. These fungal pathogens are not only detrimental to staple crops such as rice, maize, wheat, potatoes, and tomatoes but also affect a wide range of fruits and vegetables, leading to decreased agricultural productivity and food availability [2,3,4,5], which directly impacts consumers through food shortages and price fluctuations [6]. Furthermore, the resilience of these fungal pathogens to fungicides and changing climate conditions aggravate the challenge, making management and control measures increasingly difficult. The economic strain on farmers and on the broader agricultural sector signifies the urgent need for improved disease management strategies and eco-friendly approaches to safeguard economically significant food crops. The use of conventional methods such as chemical pesticides has been heavily relied on for so many years. While this is effective in the short term, these chemicals will eventually accumulate in the environment, leading to various negative drawbacks [7,8]. Most pesticides applied to plants or soil eventually disperse into the surrounding environment. Soil-applied pesticides may inadvertently spread and contaminate other areas in soil and surface water via flooding and surface runoffs [9]. Deeper soil layers and groundwaters may become contaminated through percolation [10]. Moreover, non-target organisms such as insect pollinators and the natural microbiota of the area may be disrupted due to harsh chemical residues, which can lead to broader ecological imbalances [11]. Lastly, the use of systemic fungicides, which act primarily at single target sites, has significantly increased the cases of fungicide resistance since their usage began in the 1960s. The specificity of these fungicides allows single-gene mutations to occur at the target site, leading to the development of resistance in fungal populations. As a result, over time, pathogens have adapted to the effects of these fungicides, making them less effective, contributing to the growing challenge of managing fungal diseases [12]. Thus, there is an increased demand for organic and sustainable agricultural practices for managing fungal diseases in crops. One promising approach is the utilization of biocontrol agents, which are naturally occurring organisms or substances that suppress the growth or activity of harmful pathogens [13]. Recent studies have demonstrated that numerous other microorganisms thrive inside the lichen thalli, de-establishing the long-standing belief in the dual nature of the organism. Among these microorganisms are endolichenic fungi (ELF). Endolichenic fungi refers to any fungi residing inside the lichen thallus, forming a mutualistic relationship with their hosts. The group forms a distinct lineage under the phylum of Ascomycota and Basidiomycota [14,15]. Furthermore, U’Ren et al. [16] reported that ELF constitutes a defined ecological category and is not just an incidental colonizer of lichens, highlighting their unique ecological niche within the lichen symbiotic system.

Interestingly, ELF produces an array of bioactive secondary metabolites, reported to be different from those generated by their host lichens [17,18,19,20]. These compounds exhibit various structural frameworks, such as alkaloids [21,22,23], simple nonaromatic polyketides [24], complex aromatic polyketides [25,26], polyphenyls [27], steroids [28], and terpenoids [29]. These compounds were reported to be associated with diverse biological activities that includes antibacterial [19,29,30,31,32,33], antifungal [33,34,35], cytotoxic [29,36,37], antioxidant [33,38,39], and anti-inflammatory [40,41]. Endolichenic fungi has been documented from diverse lichen hosts thriving in various ecosystems ranging from the coldest polar areas to temperate regions and are abundant in the tropics [19,31,33,38,42,43,44]. These investigations have also revealed that biotic and abiotic factors such as climate, elevation, geographical location, host lineage, and host type can influence the taxonomic diversity and occurrence of endolichenic fungal communities within its host lichen [16]. In the Philippines, only a few biological studies have utilized ELF [19,31,33,38,45,46]. However, none of these studies have specifically investigated the use of ELF as an antifungal agent against crop filamentous pathogens. Following this, it would be worthwhile to screen similar endolichenic fungi isolated from different lichen hosts growing in different habitats to test any variability in terms of secondary metabolite production and bioactivities. Therefore, in the present study, two prominent fruticose lichens in the Philippines, specifically *Ramalina* and *Usnea,* were used for the isolation of endolichenic fungi. The two lichen hosts were selected based on previous reports that demonstrated that they yield a diverse range of ELF species with bioactive properties [19,38,47,48,49,50,51]. The current study examines the antagonistic potential of ELF isolates against filamentous fungal pathogens. It also evaluates the antifungal properties of crude culture extracts from similar endolichenic fungi, which were isolated from these two lichen hosts.

## 2. Materials and Methods

### 2.1. Sampling Sites and Lichen Collection

**Figure 1:**
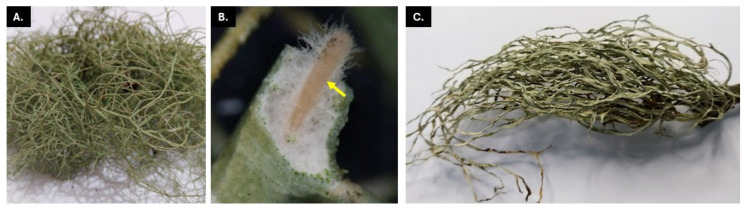
Lichen hosts: (**A**) *Usnea* sp. collected from Benguet, with thallus distinguished by its central cord (**B**), a key identifying feature of the lichen; and (**C**) *Ramalina* sp. collected from Laguna.

The lichen thalli of *Ramalina* sp. and *Usnea* sp. were collected from the provinces of Laguna (14°03′60.00″ N, 121°19′60.00″ E) and Benguet (16°54′22.79″ N, 121°03′9.00″ E), respectively (Figure 1). Both provinces are situated in Luzon Island, Philippines, and are approximately 300 km apart from each other. The former is characterized by a semi-developed residential landscape and sits at a low elevation of 118–174 m above sea level. The latter is known for its mountainous terrain and is situated in the Cordillera Mountain range with high elevation between 1266 and 1482 m above sea level which significantly influences its climate. The lichen thalli in both sampling sites were collected from barks of trees growing along the road. The average annual temperature in Laguna is approximately 26 to 28 °C while Benguet has a cooler temperature ranging from 15 to 22 °C. The tree barks were ensured for the presence of the lichens, and healthy-looking thalli were cut out from the tree bark with a knife and placed into a brown paper collection bag. The collected lichen samples were brought to the laboratory and were processed within 24 h of collection.

### 2.2. Endolichenic Fungi Isolation and Purification

The lichen thalli were rinsed in running tap water to eliminate any soil or debris. Using a sterile scalpel, the lichen thalli were cut into approximately 1–2 cm^2^ pieces. These segments were then subjected to successive washes according to the protocol of Paranagama et al. [52] with slight modifications. The segments were first immersed in 70% ethanol for 10 s, followed by submersion in 0.5% NaOCl for 3 min, and then re-immersed in 70% ethanol for 90 s. Finally, they were rinsed three times in sterilized distilled water and dried with sterile filter paper. After surface sterilization, five segments were plated onto Dichloran-Rose Bengal Chloramphenicol agar (DRBCA, Himedia, Delhi, India). The plates were incubated at room temperature for 14 days with daily monitoring. Emerging fungal hyphae were purified and transferred to Potato Dextrose Agar (PDA, TMmedia, Delhi, India) slants for storage as stock cultures. Pure endolichenic fungi was vouchered and was given a strain code.

### 2.3. Morpho-Cultural Characterization of Endolichenic Fungi

A total of forty ELF colonies were selected randomly, twenty from each of the two lichen hosts. These ELF isolates were initially cultured on PDA and then were described based on colonial growth after fourteen days or upon reaching maturity. The colonial morphologies were described based on their appearance/texture, elevation, margin, growth orientation, and color (on top and reverse). Furthermore, the fungal growth rates based on colonial diameter (expressed in mm per day, in triplicates) were measured from day 2 until day 10 of incubation. The fungal growth rates per day were then averaged and the endolichenic fungi were further classified into three categories: slow-grower (0–1 mm/day), moderate-grower (1–3 mm/day), or fast-grower (3–6 mm/day) based on the criteria established by Asthana et al. [53].

### 2.4. Co-Cultivation Assay Between Endolichenic Fungi and Fungal Phytopathogens

The test plant fungal pathogens, namely *Colletotrichum gloeosporioides* (PUPML-2020262, causative agent of anthracnose disease on pomelo), and *Cladosporium cladosporioides* (PUPML-2019252, causative agent of black mold on post-harvest tomato fruit), were obtained from the Mycology Laboratory, Engineering Science Research Center (ESRC), Polytechnic University of the Philippines (PUP) in Sta. Mesa, Manila. The strain of *Fusarium oxysporum* was obtained from a symptomatic banana. Forty ELF isolates, 20 from *Ramalina*, here designated as *Ramalina* ELF, and 20 from *Usnea*, here designated as *Usnea* ELF, were selected and then subjected to antagonistic assay against the three fungal plant pathogens. Fungal discs (approximately 5 mm in diameter) of a 7-day old plant fungal pathogen were placed 15 mm away from the edge of the PDA plate equidistant to the challenged endolichenic fungi (also, 7-day old). Control plates were also included by placing an ELF isolate or a fungal pathogen 15 mm away from the edge of PDA plates solely. The dual-culture plates were sealed and incubated at room temperature. Measurements of colonial growth expressed as radial growth were taken for 10 consecutive days [54]. The assay was conducted with three technical replicates. The colonial (mycelial) radial growth of the test fungi on a control (r1) and in the direction of the challenge plate (r2) were measured, and the percent inhibition in mycelial growth was calculated based on the formula: percent inhibition (%I) = [(r1 − r2)/r1] × 100. Furthermore, the ELF isolates were grouped based on the % inhibition with the following categories: <25%I (minimal inhibition), 25–50%I (moderate inhibition), 51–75%I (significant inhibition), and >75%I (highly significant inhibition).

The type of interaction was also recorded on day 10 following the criteria made by Wicklow et al. [55]. These include the following: (1) Interaction type A: mutual intermingling growth between the two fungi with no antagonism or inhibition of both fungi; (2) Type B: a mutual inhibition with minimal clear zone (<2 mm) upon contact or at a distance between fungi; (3) Type C: mutual inhibition at a distance; (4) Type D: inhibition of one organism on contact; the antagonist continues to grow, unchanged or at a reduced rate, through the colony of the inhibited organism; (5) Type E: inhibition of one organism at a distance and the antagonist species continuing to grow at an unchanged or reduced rate, resulting in a clear zone. Then, the observations were extended until the 15th day to check further the mycelial interaction between the two co-cultured fungi.

### 2.5. Molecular Identification and Phylogenetic Analysis

Molecular analysis was conducted to provide a more detailed characterization of the selected ELF isolates. These isolates were chosen based on their antagonistic activity, specifically those that demonstrated antagonism against the fungal pathogens used in the assay. The pure axenic samples were sent directly to Macrogen Inc. (Seoul, South Korea) for outsource DNA extraction, PCR amplification, and sequencing. The genomic DNA of the ELF isolates were then amplified using PCR with primers targeting the Internal Transcribed Spacer (ITS) region. The amplification was carried out with the forward primer ITS1 (5′-TCCGTAGGTGAACCTGCGG-3′) and the reverse primer ITS2 (5′-GCTGCGTTCTTCATCGATGC-3′) [56]. The acquired gene sequences of selected ELF isolates were analyzed for the construction of a phylogenetic tree. The gene sequences of the ELF isolates were processed and later deposited to the National Center for Biotechnology Information (NCBI) Taxonomy Database (http://www.ncbi.nlm.nih.gov/taxonomy, accessed on 11 November 2024) for the GenBank accession numbers. For the construction of phylogenetic trees, the chromatogram results of each gene sequences from the selected ELF isolates were initially evaluated with BIOEDIT. Ideally, the sequences should have no overlapping peaks and noises. The forward and reverse sequences undergo assembly using Seqman II v. 5.0. The resulting contigs were uploaded in NCBI databases via the BLAST algorithm (https://blast.ncbi.nlm.nih.gov/) with default programs to obtain closely related sequences (≥97% identity). The obtained sequences were aligned with Multiple Alignment using Fast Fourier Transform version 7.4 (MAFFT v. 7.4) [57] through TrEase web service and the construction of the phylogenetic tree was performed using RAxML software using the same web service (https://thines-lab.senckenberg.de/trease/, accessed on 2 November 2024). Bootstrap analyses and calculations were based on 1000 iterations. The generated phylogenetic trees from the analyses were viewed using MEGA v. 11. and were further edited using Microsoft PowerPoint 365.

### 2.6. SEM Analysis of Mycelial Interaction

To further examine the mycelial interactions between the co-cultured fungi, particularly between ELF and *F. oxysporum* due to its significance as a pathogen affecting a wide range of economically important crops, 15-day old cultures were used for SEM observations. Samples (mycelial plugs) for SEM observation were only performed on co-cultured fungi with close contact with each other. An agar block was cut from the interacting mycelia and observed under a scanning electron microscope (Hitachi TM3000, Tokyo, Japan) and observed for hyphal coiling of ELF over the pathogen indicating mycoparasitism or a parallel growth between the hyphae of the two co-cultured fungi further confirming mutual intermingling or absence of mycoparasitism.

### 2.7. Mycelial Growth Inhibition Assay

To evaluate the antifungal activities among similar ELF species isolated from two different lichen hosts, four ELF isolates were assessed. Specifically, two isolates identified as *Xylaria arbuscula* [*Ramalina* ELF-LS1A.4 and *Usnea* ELF-BG1D.1] and two isolates identified as *Nemania feicuiensis* [*Ramalina* ELF- LS2B.1 and *Usnea* ELF- BG4B.5.1], were grown in 200 mL potato dextrose broth (PDB, TMmedia, India) for 30 days at room temperature under static condition and then extracted with ethyl acetate. The crude culture extracts were concentrated in vacuo and reconstituted with methanol. *F. oxysporum* was selected for this assay, as it is regarded as the most severe and persistent pathogen among the three tested fungi. Following the protocol of Paguirigan et al. [58], with minor modifications, seven-day-old cultures of *F. oxysporum* were inoculated at the center of 90 mm Petri dishes and incubated for three days, allowing the mycelial growth to cover approximately half of the plate. After three days of incubation, 10 µL of crude culture extracts (50 mg/mL) were applied to 6 mm paper discs and placed around the edge of the plate. Methanol and 10 mg/mL mancozeb were used as negative and positive controls, respectively. The culture plates in triplicates were further incubated for another three days. The results were then analyzed qualitatively with the antifungal activity classified as ‘active’ when the fungal mycelia did not extend to the paper discs containing the culture extracts. It was considered ‘partially active’ if the mycelia reached but did not fully cover the paper discs; otherwise, it was inactive if the test fungi had completely grown towards the discs [58].

## 3. Results

### 3.1. Morpho-Cultural Traits of Ramalina and Usnea Endolichenic Fungi

**Figure 2:**
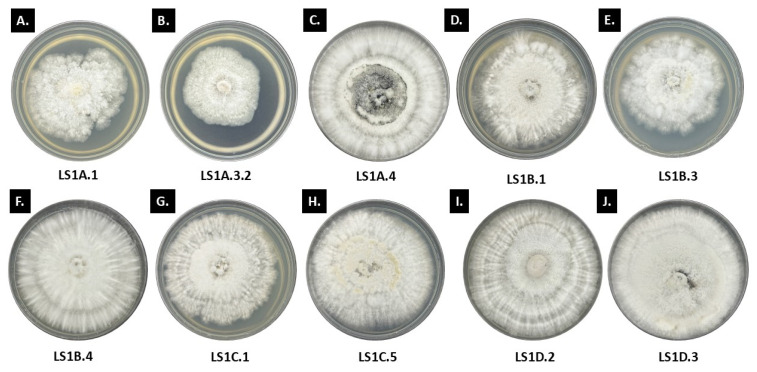

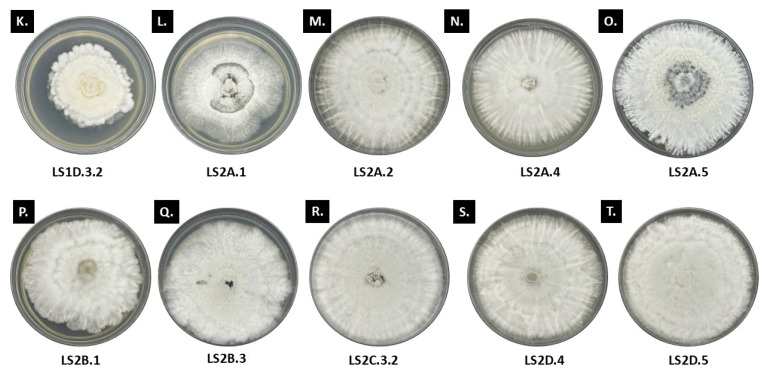
Endolichenic fungi isolated from *Ramalina* sp. grown on PDA and are incubated for 14 days under room temperature and ambient light.

Forty ELF isolates from *Ramalina* sp. and *Usnea* sp., 20 ELF from each lichen host, was characterized morpho-culturally based on their general colonial appearance. Please see Supplementary Tables S1 and S2 for the detailed colonial description of the ELF isolates. Looking closely at these 40 ELF isolates, we observed some general patterns for their colonial growth (Figure 2). Generally, the 20 ELF isolates from *Ramalina* initially showed white to cream colonies, transitioning to darker shades like brown or ash gray to black as they mature. The reverse is typically white, turning dark with prolonged incubation. Coloration was observed specially at the center or in the midpart of the colony for some isolates—LS1A.1, LS1B.3, and LS1C.5, which have clear to pale yellow exudates while LS1A.4 has black pigmentation at the center. The hyphal structure varies from semi-submerged to abundantly growing surface hyphae. For example, LS1A.1, LS1A.3.2, and LS1B.3 exhibited semi-submerged mycelia with minimal aerial hyphae, while LS2A.1 had sparsely distributed mycelia that are not submerged in the culture media. The rest of the *Ramalina* ELF isolates showed moderate to abundant aerial hyphae. The colony texture varies among the *Ramalina* ELF isolates. Some *Ramalina* ELF isolates have velvety, cottony, and powdery textures, which may suggest different survival strategies for the retention of moisture and absorption of nutrients. Many *Ramalina* ELF colonies had raised or embossed centers, which indicate an active growth pattern, while the margin characteristics were mainly filamentous, feather-like to undulate.

Furthermore, based on the colonial growth rates, most of the *Ramalina* ELF isolates were considered to exhibit moderate to fast mycelial growth per day. Fast-growing *Ramalina* ELF isolates (*n* = 8) such as LS1D.3 (=3.60 mm/day) and LS2A.5 (=3.66 mm/day) have elevated centers due to an abundant aerial mycelium. Their growth patterns are generally radial, with distinct radiating filaments and/or concentric rings, which may suggest colony expansions originating from a densely packed central area. The majority of our *Ramalina* ELF isolates showed moderate growth (n = 12), and these isolates tend to produce moderate aerial mycelia, with fluffy to cottony centers that gradually become less dense as it approaches the edge. The colony elevation is slightly raised, especially at the center, but is not distinct as compared with the fast growers. The colony margins are often irregular or undulate, with a filamentous or fimbriate appearance which produces many fine, thread-like structures. The irregular or undulate margin means that the growth is not uniform. This pattern may also suggest that the fungi are establishing themselves in the media rather than aggressively expanding, comparable to the fast-growing fungi that lean towards smoother, more uniform edges as they rapidly expand outward. There are no ELF isolates from *Ramalina* that were categorized as slow grower based on the aforementioned scheme.

**Figure 3:**
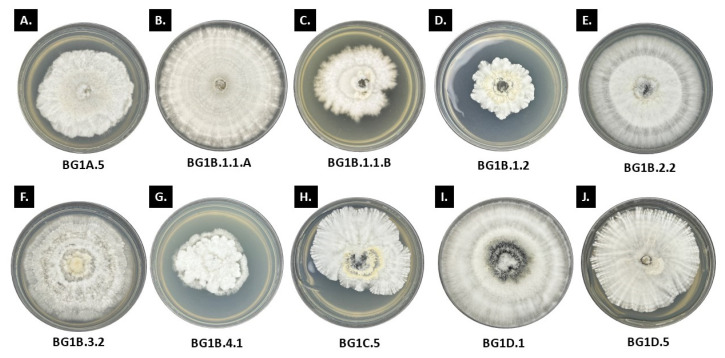

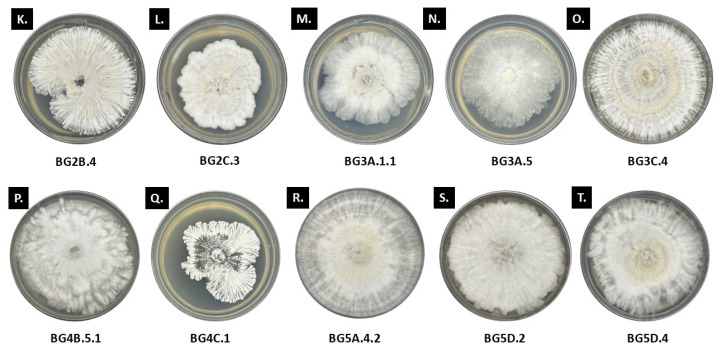
Endolichenic fungi isolates from *Usnea* sp. grown in PDA and incubated for 14 days under room temperature and ambient light.

For the 20 endolichenic fungi isolated from *Usnea*, the general colony colors were white to off-white, with varying pigmentations at the center of the culture plate (Figure 3). Most of the *Usnea* ELF isolates appeared to have feather-like mycelia that turned into darker shades of brown with some that transitioned to black as they matured. Based on their colony growth rates, the type of growth was similar with that of the *Ramalina* ELF in which the *Usnea* ELF isolates showed moderate to fast growth per day. However, it is noteworthy to mention that the growth values for *Usnea* ELF isolates were generally much lower as compared with the *Ramalina* ELF isolates. The measurements vary significantly, ranging from 1.61 mm/day to 4.60 mm/day. The *Usnea* ELF isolates like BG4B.5.1 (=4.60 mm/day) and BG1B.4.1 (=3.61 mm/day) have abundant aerial mycelia and dense cottony textures, which tend to show faster growth. Slower-growing *Usnea* ELF isolates such as BG1B.1.2 (=1.61 mm/day) and BG4C.1 (=1.68 mm/day), tend to have more minimal, compact, or diffuse mycelia, which may signify a more energy-conservative growth strategy.

Since ELF isolates from the two lichen hosts did not produce spores even on prolonged incubation, it would require the use of molecular methods to confirm identities. Based on their prevailing morpho-cultural traits, the majority of the isolated endolichenic fungi tends to belong to the family Xylariaceae due the feather-like filamentous edges and production of distinct stroma.

### 3.2. Interactions Between Endolichenic Fungi and Test Fungal Phytopathogens

#### 3.2.1. Antagonistic Activity of *Ramalina* ELF Isolates

Based on the results, the endolichenic fungi isolated from the fruticose lichen *Ramalina* reduced the growth of the three plant fungal pathogens at various degrees, showing their potential as biocontrol agents (Table 1). The *Ramalina* ELF isolates significantly inhibited the growth of *C. gloeosporioides* with the range of 32.39% ± 2.57 to 57.78% ± 2.57. The ELF isolate LS2B.3 inhibited the fungal pathogen the most, achieving the highest inhibition rate of 57.78% ± 2.57 on the 10th day. Moreover, five *Ramalina* ELF isolates exhibited at least 50% inhibition of *C. gloeosporioides*, and this included isolates LS2A.5 (55.47% ± 3.68), LS2B.1 (51.45% ± 7.44), LS1A.4 (50.94% ± 2.74), LS1D.2 (50.85% ± 5.85), and LS1D.3.2 (50.09% ± 1.04). Consequently, *Ramalina* ELF LS2A.1 had the lowest activity amounting for only 32.39% ±2.57. Conversely, most *Ramalina* ELF isolates demonstrated moderate activity, with all having inhibition rates of >32%. Nevertheless, our antagonistic assay revealed that all 20 *Ramalina* ELF isolates inhibited the mycelial growth of *C. gloeosporioides*, though the degree of inhibition varied between isolates (Table 1). See also Supplementary Figures S1–S6 for images of the dual culture assays.

**Figure 4:**
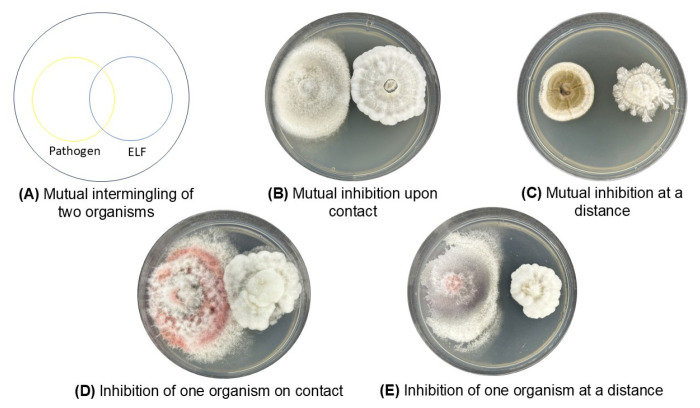
Antagonistic activities of test fungi against the plant pathogens.

The type of interaction among the *Ramalina* ELF isolates against *C. gloeosporioides* was also observed (Table 1; Figure 4). The Type B interaction was observed on isolate LS1A.4, where there was almost mutual inhibition upon contact between the two interacting fungi, and the space between the isolates on the time of the observation was small but was clearly marked. The percentages of the inhibition of growth of the two interacting fungi were almost the same, that is, 52.65% ± 2.23 and 50.94% ± 2.74, which explain the mutual reduction in their growth. Moreover, only isolate LS2A.1 had Type C interaction. This isolate was also inhibited by the pathogen at almost the same rate (33.81% ± 6.60), proving that they were mutually inhibiting each other. Furthermore, a total of 15 *Ramalina* ELF isolates were recorded to exhibit Type D interactions. Notably, only seven of these *Ramalina* ELF isolates demonstrated a higher inhibitory percentage activity than that of *C. gloeosporioides*, suggesting that these endolichenic fungi possess greater inhibitory activity than the fungal pathogen. These isolates tend to have a growth trend in which, following direct contact, the endolichenic fungi continued to grow on the surface of the pathogen, which subsequently fully inhibited the growth of the pathogen over time. Interestingly, some of these isolates are putatively identified as belonging to the family Xylariaceae due to their prominent radiating mycelia and feather-like edges. Conversely, eight *Ramalina* ELF isolates within this interaction type had lower inhibitory activities than the fungal pathogen. These isolates include LS1B.3, LS1B.4, LS1C.5, LS2A.2, LS2A.4, LS2B.1, LS2C.3.2, and LSD2.5, indicating that after 10 days, the fungal pathogen prevailed in the assay. Furthermore, there were three isolates that were observed to have Type E interaction, and this includes isolates LS1A.1, LS1A.3.2, and LSD2.4. However, all three of these plates have the pathogen dominating over the endolichenic fungi.

The twenty *Ramalina* ELF isolates were also screened against *C. cladosporioides*, a causative agent causing black mold on post-harvest tomato fruit. Interestingly, the inhibitory activity of the endolichenic fungi from *Ramalina* against *C. cladosporioides* was the lowest as compared to the other two fungal pathogens, having the range of 15.67% ± 8.94 to 47.21% ± 1.70. For most of the isolates, the endolichenic fungi exhibited higher inhibitory activity and resistance against *C. cladosporioides*, demonstrating the potential of this group against the pathogen. The isolate LS2A.2 had exhibited the highest inhibitory percentage with 47.21% ± 1.70, while LSD2.5 was recorded to be the lowest with 15.67% ± 8.94. Most of the *Ramalina* ELF isolates had >30% inhibitory activity but were not able to exceed 50%. Despite this, most of the endolichenic fungi were able to resist the effects of the pathogen towards them, showing the potency of the isolates in controlling the pathogen. Based on the interactions observed after 10 days, it is evident that most of the *Ramalina* ELF isolates exhibited Type D interaction against *C. cladosporioides* while only three ELF isolates had Type E interaction, and this includes isolates LS1A.1, LS1A.3.2, and LS2A.1. The rest of the endolichenic fungi were able to inhibit *C. cladosporioides* through direct contact (Type D). Most of the *Ramalina* ELF isolates, as observed with the other fungal pathogens, halt the growth of *C. cladosporioides* after contact. A general pattern can be inferred that a gradual decrease in the growth of the pathogen was observed before they completely stopped growing, which is observed when the samples were incubated further for 15 days. Some of the *Ramalina* ELF isolates growth, which includes LS1B.3, LS1B.4, LS1C.1, LS1D.2, LS1D.3.2, and LS2B.3 were observed to have been stimulated, which is indicated by negative inhibition. Growth stimulation means that some endolichenic fungi had recorded an increase in their radial growth in the plate in the presence of the pathogen as compared to their single culture (=control plate).

**Figure 5:**
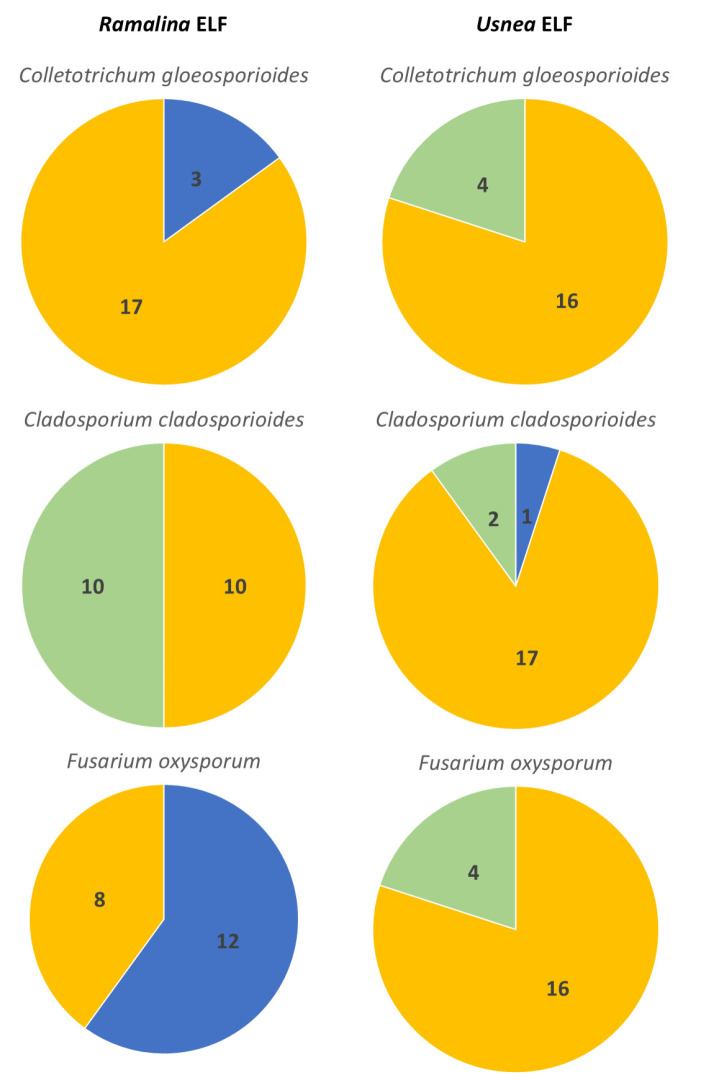
The number of ELF isolates based on their % inhibition of the three plant pathogens. The categories are described as follows: <25%I (minimal inhibition), 25–50%I (moderate inhibition), 51–75%I (significant inhibition), and >75%I (highly significant inhibition). A total of 20 ELF from each of the two lichen hosts were assessed for each plant fungal pathogens. Note: <25% (Green), 25–50% (Yellow), 51–75% (Blue), >75% (Red).

Lastly, the ability of endolichenic fungi to inhibit *F. oxysporum* that was isolated from a symptomatic banana was also evaluated. The highest inhibitory percentage against *F. oxysporum* was recorded with *Ramalina* ELF isolate LS2B.1 with 61.44 ± 3.87, while the lowest was observed in *Ramalina* ELF isolate LS1A.3.2 having 34.21 ± 5.16. Compared with the activity of the endolichenic fungi against the two other pathogens, the inhibitory percentage that was collated against *F. oxysporum* was higher as opposed to the other two pathogens. Of the 20 *Ramalina* ELF isolates, 13 ELF isolates achieved a >50% inhibitory activity against the test pathogen, but at the same time, it is also worth noting that the resistance of the endolichenic fungi against the pathogen were close with the percent inhibition. Nevertheless, the growth of isolate LS2D.5 was induced by the interaction assay. The present study served as an effective basis in screening endolichenic fungi that can be further processed to isolate potential bioactive compounds. Two *Ramalina* ELF isolates, namely LS2A.5 and LSD2.4, exhibited Type B interaction, that is, both cultures were mutually inhibited upon contact. Most of the isolates showed Type D interaction; nine isolates had low resistance against *F. oxysporum*, while eight isolates successfully resisted and were able to dominate the pathogen in the interaction. Only one isolate, LS1A.3.2, exhibited Type E interaction, where the pathogen had inhibited the endolichenic isolate at a distance. The number of *Ramalina* ELF isolates based on their percent inhibition of the three plant pathogens is presented in Figure 5.

#### 3.2.2. Antagonistic Activity of *Usnea* ELF Isolates

We also evaluated 20 ELF isolates from *Usnea* for antagonistic activity as similarly conducted with *Ramalina* ELF isolates. Based on the results, a considerably lower inhibitory activity was calculated with the *Usnea* ELF isolates as compared with *Ramalina* ELF isolates. Nevertheless, the *Usnea* ELF isolates were still able to reduce the growth of the pathogens in variable degrees (Table 2; Figure 4). See also Supplementary Figures S1–S6 for images of the dual culture assays.

Against *C. gloeosporioides*, the *Usnea* ELF isolates were not able to achieve an inhibitory activity exceeding 50%, as shown in Table 2. Among them, BG1B.1.2 and BG1D.5 showed the highest activity with both exhibiting a percent inhibition of 45.56% ± 10.53 and 45.56% ± 3.84, respectively. In contrast, the lowest inhibition was recorded for isolate BG1B.3.2, which showed only 18.80% ± 1.84. The morphological characteristics of the pathogen remain relatively unaffected across different interactions. This is also the same with the endolichenic fungi where most of the isolates remained white to off-white in color; however, a significant exclusion is the isolate BG3C.4, which has a noticeable production of a more vibrant curry-yellow to orange yellow pigmentation as compared with the control plate. This may indicate the possible secondary metabolite production as a stress response of *Usnea* ELF, particularly when they interact closely with *C. gleosporoides*. In some instances where the two fungi meet, the colony edges display morphological changes, including denser mycelial structures that form a noticeable white line. This boundary alteration may also be a stress-induced response, reflecting the defense mechanism at the interaction zones. The type of interaction observed on the 10th day is dominated by Type E interaction, and this is maybe because the *Usnea* ELF isolates have relatively slower growth per day as compared with the pathogen. However, some endolichenic fungi were able to fully inhibit the growth of the pathogen after contact. Several endolichenic fungi under this interaction exhibited higher inhibitory activity than that of the pathogen, which means that the growth that was inhibited on the pathogen was greater than that of the endolichenic fungi. These isolates include BG1B.1.2, BG1C.5.1, and BG3A.1.1. On the other hand, only two isolates had Type B interaction, and these are isolates BG1B.1.1. B and BG1B.2.2. The other two tested pathogens were observed to display variations among color and growth patterns in our dual culture assay. Interestingly, the *Usnea* ELF isolates have demonstrated a higher inhibitory activity against *C. cladosporioides* in comparison with the *Ramalina* ELF isolates. A common pattern for the pathogen *C. cladosporioides* was that it showed darker brown to black pigmentation on culture plates, which present a clearance zone between it and the *Usnea* ELF isolates—BG1A.5, BG1B.1.2, BG1B.4.1, BG1D.1, and BG4C.1. An exclusion is with the isolate BG5D.4; however, it still has a minimal space between the interacting fungi. Interestingly, prolonged incubation of the dual cultures showed that most of the endolichenic fungi have still been able to grow on the surface of the pathogen, including those plates with *C. cladosporioides* displaying melanin production. The Type D interaction was also observed with isolates BG1B.1.1. B, BG1B.2.2, BG3C.4, BG5D.2, and BG5D.4. All of them were able to record higher inhibitory activity against the pathogen. Only isolate BG1B.1.2 showed a Type C interaction. Lastly, the *Usnea* ELF isolates were also paired with the pathogen *F. oxysporum*. Just like the previous pathogens, the presence of antagonists or competing organisms may induce stress responses in *F. oxysporum*, potentially altering its morphological characteristics. The pathogen exhibited varied traits when paired with different endolichenic fungi, which may result from these differing interactions. In most cases, the pathogen demonstrated a competitive advantage in the assay, successfully overcoming the challenges posed by the endolichenic fungi. Nevertheless, there are endolichenic fungi that have successfully inhibited the pathogen, and these *Usnea* ELF isolates include BG1B.3.2, BG1B.4.1, BG2C.3, and BG4B.5.1. Unfortunately, no *Usnea* ELF isolate exceeded 50% inhibitory percentage. The highest inhibitory activity was recorded from the isolate BG5D.4, which has only 43.30% ± 0.29, while the lowest was from BG3A.5 with 13.20% ± 13.38. Type D interaction prevailed among the assays. Most of the pathogens exhibited denser mycelial growth in the interaction zone, with an almost completely submerged center. This denser mycelium likely serves as a protective mechanism against the endolichenic fungi, indicating that the pathogens allocate their energy towards producing this robust mycelial structure for defense. Figure 5 also shows the number of *Usnea* ELF isolates based on their percent inhibition of the three plant pathogens.

### 3.3. Molecular Identification and Phylogenetic Analysis of Selected Endolichenic Fungi

**Figure 6:**
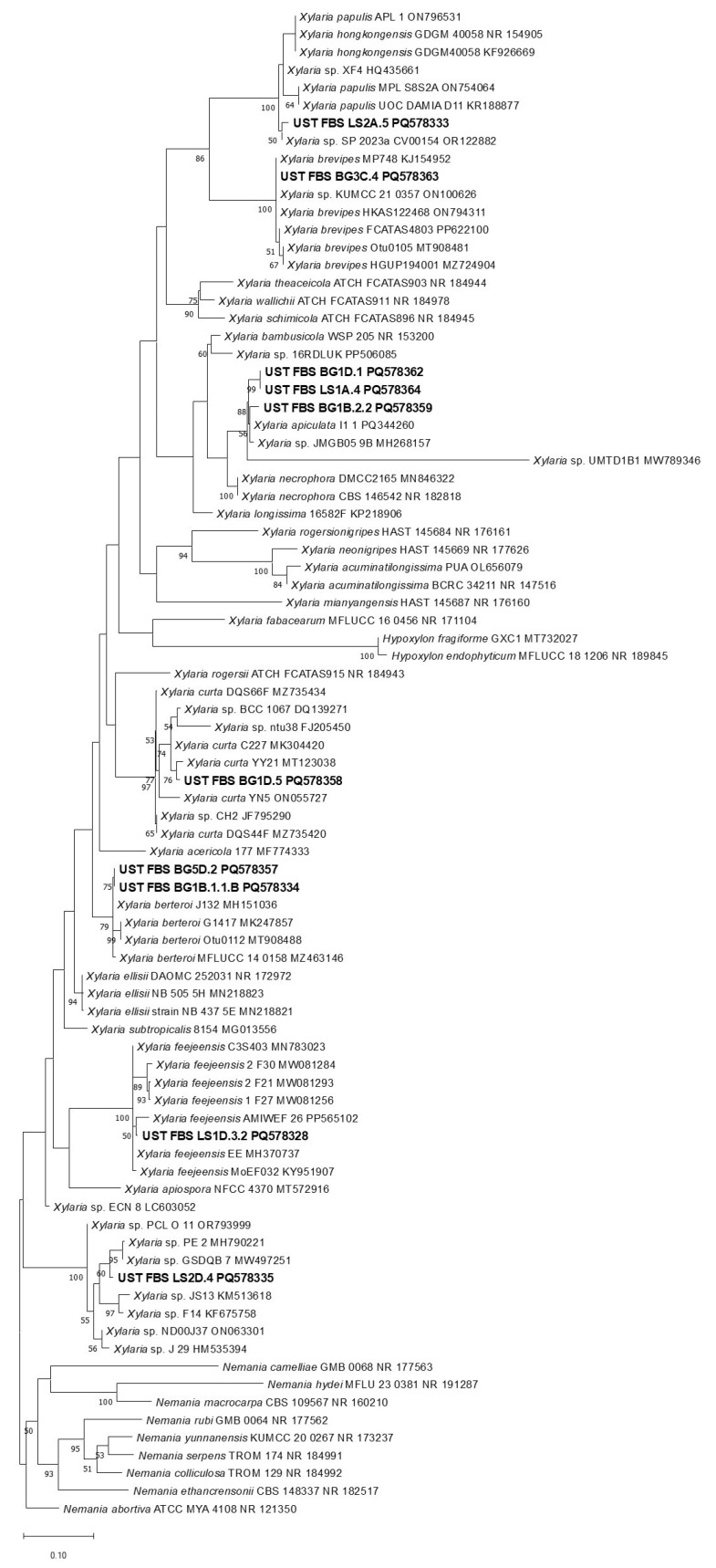
Phylogenetic tree of ELF isolates from *Ramalina* and *Usnea* initially identified belonging to genus *Xylaria* based on the tree inferred with RAxML, having 1000 generations (bootstrap).

All *Ramalina* ELF isolates generally exhibited significant inhibition (>50%I) in at least one of the test fungi, while the *Usnea* ELF isolates achieved different levels of inhibitory activities, i.e., moderate (<50%I) to significant (>50%I) inhibition. From these, we selected 15 ELF isolates (six *Ramalina* ELF, nine *Usnea* ELF), which had exhibited at least moderate inhibition for the phylogenetic analysis to check for their identities. Based on the constructed phylogenetics trees, all of the identified ELF isolates belong to the family Xylariaceae. The ELF isolates identified from the two lichens showed to be congeneric with each other. Further analysis of our results showed that four *Ramalina* ELF and six *Usnea* ELF isolates were identified as belonging to the genus *Xylaria* (Figure 6). The isolates LS1A.4, BG1D.1, and BG1B.2.2 formed close association with *Xylaria apiculata* PQ344260 associated with spalted wood pieces in a premontane forest of Peru [59] and *Xylaria* sp. JMGB05 9B (MH268157), which was identified as an endophyte of Amazonian hardwood tree [60].

The isolate LS1D.3.2 had formed a distinct clade with *Xylaria feejensis* with closest sequence *X. feejeensis* AMIWEF—26 (PP565102), which was isolated as endophyte from *Aquilaria malaccensis.* Moreover, *Usnea* ELF BG1B 1.1.B and BG5D.2 formed a clade with *Xylaria berteroi* with some isolates identified as fungal endophytes from China and Thailand. The isolate BG1D.5 paired with *Xylaria curta* YY21 (MT123038) reported to be a fungal endophyte in grapevines. Isolate BG3C.4 had grouped with *Xylaria brevipes*. Lastly, the two other ELF isolates have broadly formed a group with other fungi. The ELF isolate LS2A.5 had paired with a fungal isolate *Xylaria* sp. SP-2023a-CV00154 (OR 122882) reported as cave mycobiota in Thailand [61].

Subsequently, isolate LS2D.4 grouped together with *Xylaria* sp. PE2 (MH790221), which was identified as fungal endophyte from India and with *Xylaria* sp. GSDQB 7 (MW497251), a pathogen causing leaf spots. From this, it can be illustrated that the endolichenic fungi had a wide range of possible lifestyles in its life cycle.

**Figure 7:**
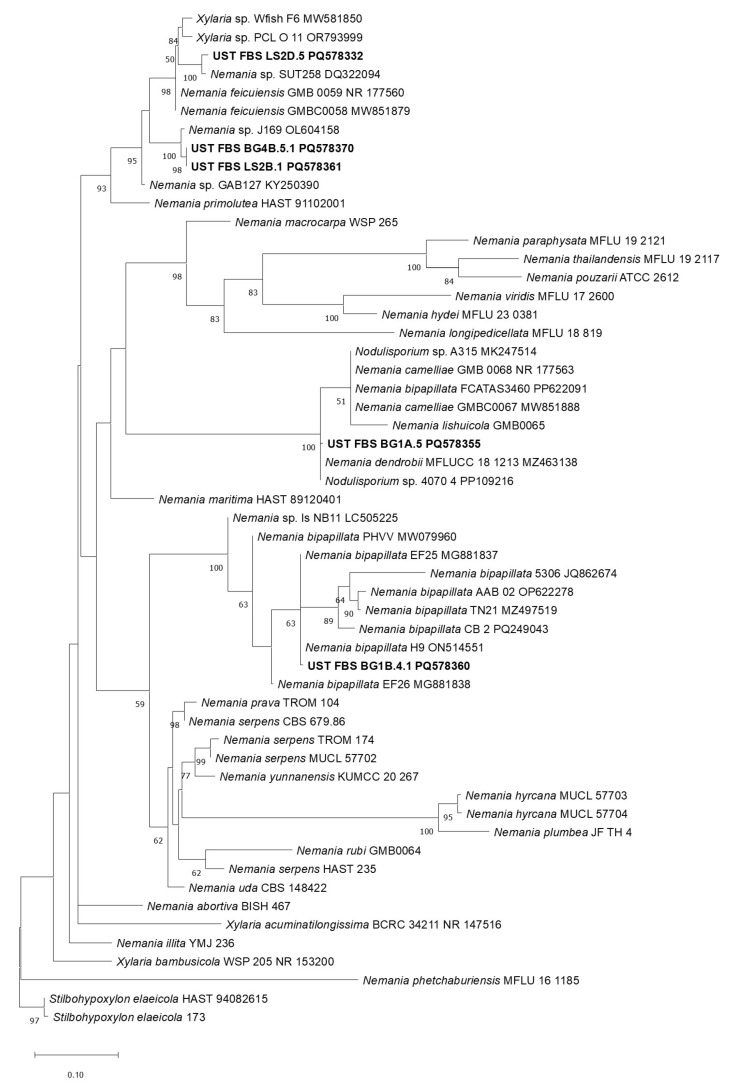
Phylogenetic tree of ELF isolates from *Ramalina* and *Usnea* initially identified as belonging to genus *Nemania* based on the tree inferred with RAxML, having 1000 generations (bootstrap).

On the other hand, five ELF isolates were described as belonging to the genus *Nemania* (Figure 7). Two isolates from *Ramalina*, which includes LS2D.5 had formed a separate clade with *Nemania* SUT258 (DQ322094) supported by strong bootstrap value, and this strain was isolated from Thailand and LS2B.1, which paired with *Usnea* ELF BG4B.5.1, had formed a group with *Nemania* sp. J169 (OL604158). The remaining *Usnea* ELF isolates, BG1B.4.1, formed a group distinctly with *Nemania bipapillata* H9 (ON514551), identified as postharvest pathogen of dragon fruits in Guizhou, China [62], and isolate BG1A.5 formed a clade with *Nemania* and *Nodulisporium*.

### 3.4. SEM Observations

**Figure 8:**
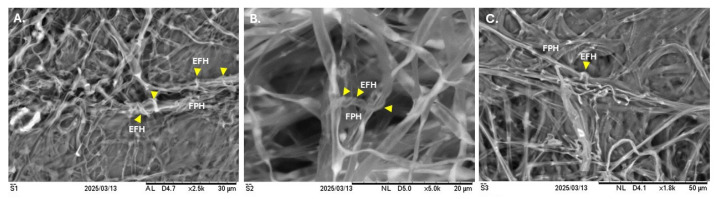
Mycoparasitism exhibited by selected ELF isolates as a mechanism to antagonize the growth of *Fusarium oxysporum*. The coiling behavior of *Ramalina* ELF—LS2A.5 (**A**) and LS2B.1 (**B**) around the mycelia of *F. oxysporum* is shown. Additionally, the *Usnea* ELF BG3C.4 (**C**) was observed wrapping around the hyphae of *F. oxysporum*. **Note**: *EFH*—Endolichenic Fungi Hyphae (which was pointed with the yellow arrow) and *FPH*—Fungal Pathogen Hyphae.

After 10 days of incubation, the number of ELF exhibiting the different categories based on percent inhibition is presented in Figure 5. To further assess these interactions, dual cultures of ELF isolates and fungal pathogens were incubated for an extended period of five days beyond the standard assay duration. This prolonged incubation allowed for the observation of subtle phenotypic differences, particularly after hyphal contact between isolates. Initial growth patterns showed that due to their relatively slower growth rates, ELF isolates appeared dominated by the rapidly expanding pathogen, as reflected in the calculated percent inhibition of growth at day 10. However, upon hyphal contact, many ELF isolates—especially those displaying feather-like colony edges—began to grow over the surface of the pathogen (Figure 4D). This observation prompted the extension of the incubation period to determine whether the ELF isolates could eventually overcome the pathogen’s initial dominance. Scanning electron microscopy revealed the mycoparasitic behavior exhibited by selected ELF isolates against *F. oxysporum* (Figure 8). The interaction between ELF isolates and the pathogen was characterized by the coiling and wrapping of ELF hyphae around the hyphae of *F. oxysporum*, suggesting a physical antagonistic mechanism. Specifically, the *Ramalina* ELF isolates LS2A.5 (Figure 8A) and LS2B.1 (Figure 8B) demonstrated distinct coiling patterns, with ELF hyphae tightly encircling the pathogen’s hyphae. Similarly, the *Usnea* ELF isolate BG3C.4 (Figure 8C) exhibited comparable wrapping behavior, further supporting the role of mycoparasitism in inhibiting *F. oxysporum* growth. Most of the *Ramalina* and *Usnea* ELF isolates demonstrated moderate antifungal inhibition.

Our results also revealed that several ELF isolates grew over and coiled around the hyphae of *F. oxysporum*, suggesting that growth suppression was not solely due to faster pathogen proliferation, but also due to direct contact inhibition and potential mycoparasitism. Additionally, evidence of antibiosis was observed in some interactions, indicated by the trimmed-like, suppressed growth appearance of the pathogen in certain plates (Figure 4C,D), suggesting the release of antifungal secondary metabolites by ELF isolates. Collectively, these findings suggest that ELF isolates employ multiple antagonistic strategies—including mycoparasitism, direct contact inhibition, and antibiosis—to suppress the growth of *F. oxysporum*.

### 3.5. Inhibition of Mycelial Growth by Selected Endolichenic Fungi

**Figure 9:**
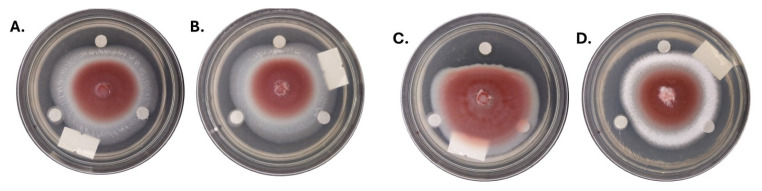
Antifungal activities of culture extracts of (**A**,**B**) *Xylaria arbuscula*; LS1A.4 and BG1D.1 and (**C**,**D**) *Nemania feicuiensis*; LS2B.1 and BG4B.5.1. Paper disks containing culture extracts (30 µL) of the endolichenic fungi were placed on the top side of the plates. Left lower disks containing a positive control (mancozeb; 10 mg/mL), right lower disks containing a negative control (methanol).

The antifungal activity of culture extracts from four ELF isolates was evaluated against *F. oxysporum* using the disk diffusion assay (Figure 9). *F. oxysporum*, which is recognized as the most widespread and persistent pathogen among the three fungal pathogens tested in the present study and is known for causing wilt diseases in various economically significant crops in the country, was used in the assay. In all assay plates, *F. oxysporum* was inoculated at the center, while paper disks containing 30 µL of ELF extracts were positioned on the upper side of the plates. Positive control disks containing mancozeb were placed at the lower left, and negative control disks containing methanol were positioned at the lower right. The ELF isolates tested included *Xylaria arbuscula* LS1A.4 (Figure 9A) and BG1D.1 (Figure 9B), and *Nemania feicuiensis* LS2B.1 (Figure 9C) and BG4B.5.1 (Figure 9D). Notably, a clear zone of inhibition was observed around the disk containing the extract of LS2B.1, indicating significant antifungal activity. This suggested that as-yet-unknown antifungal compounds in the ELF culture extracts can be used to control fusarium wilt. In contrast, LS1A.4, BG1D.1, and BG4B.5.1 showed comparatively weaker inhibitory effects, with smaller or less distinct zones of inhibition. These results suggest that ELF isolates exhibit varying degrees of antifungal activity, which may be influenced by the specific lichen host or strain variation.

## 4. Discussion

Most of the ELF isolates in the present study were described as *mycelia sterilia* due to the absence of spores. Despite various attempts to induce sporulation, including exposure to ultraviolet light to induce stress, alternating light and dark conditions, total darkness, minimal nutrient media, and extended incubation periods, the ELF isolates remained unable to produce spores. This corroborates with many previous studies wherein *mycelia sterilia* were a major proportion of endolichenic fungi isolated for all the studied lichen hosts. In the study of Tripathi et al. [63], the colonization frequency of *mycelia sterilia* were highest, reaching up to 47%, while another study [64] reported 33% occurrence rate. Additional reports by several studies [65,66,67,68] further documented the prevalence of *mycelia sterilia* among their studied lichen hosts which included *Bulbothrix setschwanensis*, *Parmotrema reticulatum*, *Flavoparmelia caperata*, *Flavopunctelia flaventior*, *Cryptothecia* sp., and *Usnea* sp. Interestingly, some ELF isolates produced pigmentation after prolonged incubation, though they still did not produce spores. Some isolates that were putatively characterized as belonging to the family Xylariaceae produce stromata, albeit without any observed spores. In the report by Cañón et al. [66], three *Xylaria* species isolated from the thalli fragments of *Cladonia curta* took longer incubation periods for the emergence of stromata, i.e., ten months for their isolates MS1 and LB1 and one year with consecutive subcultures for their isolate MS2. Most of the observed morphological traits common among the members belonging to Xylariaceae [66].

Based on the presented results, *Ramalina* ELF isolates exhibited higher inhibitory activities than *Usnea* ELF isolates. It was reported by several papers that both biotic and abiotic factors influenced the composition and properties of the endolichenic fungi [67]. Depending on the distinct habitats of the host lichens, the inhabiting endolichenic fungi might lead to variance in metabolite expressions. The sampling sites of the two lichens were very different from each other. The fruticose lichen *Ramalina* was collected mainly along the roads of San Pablo City in Laguna at a lower elevation and the area is characterized by a semi-urban landscape with an average annual temperature of approximately 26 to 28 °C. The area has visible human interventions and considerable presence of air pollution. This sampling area was also similar to the sampling site of Galinato et al. [38], in which they identified the antioxidant properties of the endolichenic fungi inhabiting the same lichen. The aforementioned study hypothesized that the presence of air pollution and stress in the environment can force these organisms to upregulate their natural defense mechanism against external stressors. When these defenses are exhausted, fungal secondary metabolites act as an adaptive response to stress conditions by scavenging reactive oxygen species. Therefore, from this it may also be inferred that the endolichenic fungi may also induce other secondary metabolites such as antimicrobial substances that are necessary for the survival of their host lichen during stressful conditions. This suggests that the environment in which a lichen thrives can indirectly influence the endolichenic fungi, shaping their responses and production of vital metabolites that increases host’s resilience. In contrast, the sampling site for *Usnea* in Benguet is characterized by a cooler temperature ranging from 15 °C to 23 °C, due to the high elevation of the region. Benguet experiences substantial annual rainfall, averaging around 3000 mm, and is often blanketed in fog and mist, particularly during the rainy season and this creates higher humidity and frequent cloud cover at higher altitudes. These conditions foster the development of montane and mossy forest ecosystems, providing a suitable habitat for *Usnea*. The evident environmental differences between the two sampling sites may account for the greater activity observed in the endolichenic fungi associated with *Ramalina* as compared to those associated with *Usnea*. In addition to air pollution, endolichenic fungi must resist a variety of harsh environmental factors inclined with the habitat of the lichen. These may include inconsistent supply of water and nutrients, limited aeration, and invariable fluctuations in temperature and relative humidity. In more extreme environments, such as deserts, these challenges are amplified by excessive exposure to UV radiation and persistent elevated air temperatures [68]. It can be inferred that the endolichenic fungi may have the ability to construct a plethora of secondary metabolites, potentially due to the evolution of novel genes that may have been triggered in response to the selection pressures from the various abiotic and biotic factors [69,70]. This concept coincides with the assumption of the present study in which the environment of the host lichen plays a significant role in influencing the production of secondary metabolites [67,71].

Despite the observed differences, it can also be inferred that most endolichenic fungi, regardless of their host origin, were able to inhibit or reduce the growth of fungal pathogens to varying degrees, particularly upon contact. Lichens are known for their ability to produce various antimicrobial compounds [72,73,74]. However, some microbial communities within lichen tissue are still able to thrive and propagate despite the presence of these antimicrobial agents [75]. This suggests that the ELF has the capacity to resist various secondary metabolites produced and excreted by their lichen host. Thus, the relationships among lichen and its inhabitants likely involve a more complex interaction that enables some microbes to persist despite the presence of antimicrobial compounds. The reason can be that lichens allow the growth of these microbes because they can also produce abundant secondary metabolites, which can help the host to resist stresses in the environment. This highlights the potential value of the ELF as a source of active biological compounds, particularly antifungal agents. In the study by Cernava et al. [76], which analyzed the antagonistic activity of bacterial species within the tissue of the lichen *Lobaria pulmonaria* (L.) Hoffm., they hypothesized that even though lichens produce various secondary compounds with antagonistic effects [77,78,79], only a diverse and protective microbiome can efficiently maintain stability over longer periods to prevent pathogen attacks. This could mean that the present endo-microbes, like endolichenic fungi can serve as a line of defense that can help sustain the production of vital metabolites in time where the host has a short supply of needed metabolites. This underscores the significant impact and potential of endolichenic fungi within the lichen response system. Moreover, Cernava et al. [76] reported that the bacterial taxa with lower occurrences in the lichen thallus predominantly acts as a defense against biotic disturbances only while those identified as highly diversified bacterial microbiome strengthen the available functional repertoire, which might play a crucial role for the stability and overall survival of the host. Since the endolichenic fungi has the same niche as the bacterial isolates examined in the previous study, it can also be assumed that this concept can also be true to the endolichenic fungi. In the present study, most of the culturable identified fungal isolates belong to the family Xylariaceae that in turn exhibited active antagonistic activity against the pathogens, proving that they are also responsible for the overall survival of the lichen hosts. Furthermore, other studies [58,68,80,81,82] have demonstrated the potential of ELF against crop fungal pathogens. However, similar studies are currently lacking in the Philippines. Therefore, the findings of this study provide initial data on ELF research in the country.

Moreover, following incubation for 10 days, the ELF isolates were further incubated for an additional five days to identify any subtle phenotypic differences, particularly between isolates that had been in contact. This extended incubation helped reveal changes in growth patterns and competitive interactions that may not appear within the standard 10-day period. A general observation was that, due to their relatively slower growth, endolichenic fungi initially appeared to be dominated (or may appear “defeated”) by the pathogen, leading to the assumption that the pathogen had prevailed. This outcome was consistent with the percent inhibition of growth calculated on day 10, showing a higher rate of growth suppression in the endolichenic fungi due to the faster growth of the fungal pathogen. However, upon contact, many endolichenic fungi, especially those with feather-like edges, started to demonstrate the ability to grow over the surface of the pathogen. Thus, the extended incubation of dual cultures could determine if the pathogen could maintain its dominance or if the endolichenic fungi, especially after contact, could start to outgrow the pathogen. Based upon our observation, most endolichenic fungi were able to grow within the surface of the fungal pathogens. Thus, the study concludes that growth suppression among the fungal pathogens resulted from direct contact inhibition, consistent with previous reports. Peters et al. [83] observed those 151 fungal endophytes inhibited the growth of *C. gloeosporioides* through various antagonistic modes, including antibiosis, competition and mycoparasitism. Notably, the most active isolates primarily exerted their effects through direct contact. Their research highlighted *Ramichloridium* sp. as an excellent candidate for bioformulations due to its rapid growth and abundant spore production. This study suggests that the suppression of *C. gloeosporioides* may arise from competition for space and nutrients, driven by the fast growth of these fungi. In contrast, the present study documented fungal isolates that are relatively slow-growing and are *mycelia sterilia*, meaning they do not produce spores in the medium, unlike the *Ramichloridium* sp. from earlier research. This distinction may explain the percent inhibition results observed on the 10th day, where many current isolates showed greater reduction in growth as compared to the pathogen. However, observations from prolonged incubation indicated that most ELF isolates ultimately outgrow the pathogen following direct contact. Most of the identified active endolichenic fungi isolates belong to the genus *Xylaria*. The members of the genus had been documented to produce lytic enzymes, such as chitinases and β-1,3-glucanases, which can degrade the cell wall of the pathogen and allow these fungi to colonize the surface of the phytopathogen colony. Similar data are reported for the antagonism between the endophyte *Xylaria* sp. isolated from mangrove against the phytopathogen *Fusarium solani* [84]. The isolates have also been observed to have mycoparasitism ability. A coiling mechanism was observed on the hyphae of these pathogenic fungi that was confirmed through light and scanning electron microscopies. In some of our ELF isolates, it is evident that there is abundance in aerial mycelia around the interaction zone. The endolichenic fungi were densely coiled around the mycelia of the pathogen. They grew along the pathogenic fungal mycelia, mostly branching and coiling around them, which resulted in possible mycelial deformities.

These observations align with the antifungal assay results of the ELF crude culture extracts. The study further determined whether similar ELF species isolated from different lichen hosts exhibit variations in the antifungal activity of their crude culture extracts. The antifungal assay demonstrated varying inhibitory effects of ELF culture extracts against *F. oxysporum*, a major pathogen causing wilt in economically important crops. Notably, *N. feicuiensis* (LS2B.1) from the lichen *Ramalina* sp. showed significant antifungal activity, indicated by clear zones of inhibition and, hence, designated as active. This suggests the presence of potent antifungal metabolites with potential for biocontrol applications. In contrast, *X. arbuscula* (BG1D.1) and *N. feicuiensis* (BG4B.5.1), both from the lichen *Usnea* sp., and *X. arbuscula* (LS1A.4) from the lichen *Ramalina* sp., exhibited weaker inhibition, designated here as partially active, as the mycelial growth reached the disks containing the crude culture extracts but did not extend over the disks. These highlight the possible strain-specific differences and/or by the influence of the lichen host. The consistency of the positive (mancozeb) and negative (methanol) controls supports the reliability of the results. But as the study only compares four isolates, this merits further studies. Nevertheless, *N. feicuiensis* LS2B.1 warrant further investigation to characterize their bioactive compounds for the development of possible agro-chemicals with potential as sustainable antifungal agents.

## 5. Conclusions

The present study investigated and compared the antagonistic activity of endolichenic fungi isolated from two fruticose lichens—*Ramalina* and *Usnea*. Our results revealed that the majority of the isolated ELF belong to the family Xylariaceae, particularly under the genera *Xylaria* and *Nemania*, and this was supported by molecular methods and morphocultural characterization. Our findings also support the notion that the lichen host and the inhabiting endolichenic fungi may have the potential to influence each other, especially under stressful environmental conditions. However, further studies are needed to provide additional evidence to support these concepts with the *Ramalina*- and *Usnea*-associated endolichenic fungi. Nevertheless, all ELF isolates successfully reduced the growth of the three filamentous fungal pathogens to varying degrees, with direct contact inhibition being the predominant trait among the endolichenic fungi. This shows the potential of ELF as eco-friendly biocontrol agents for sustainable agriculture.

## Figures and Tables

**Table 1:** Antagonistic activity of twenty *Ramalina* ELF against the three plant fungal pathogens. The type of fungal interaction for each isolate against each pathogen and the percentage of growth inhibition (I%) ± standard deviation (SD) was indicated.

Isolate Codes	*C. gloeosporioides*		*C. cladosporioides*		*F. oxysporum*	
	Percent Inhibition (I%) ± SD	Type of Interaction	Percent Inhibition (I%) ± SD	Type of Interaction	Percent Inhibition (I%) ± SD	Type of Interaction
LS 1A.1	36.50 ± 4.88	E	22.96 ± 8.88	E	47.68 ± 5.66	D
LS1A.3.2	37.35 ± 8.68	E	28.11 ± 13.23	E	34.21 ± 5.16	E
LS1A.4	50.94 ± 2.74	B	35.19 ± 3.55	D	49.62 ± 4.62	D
LS1B.1	38.29 ± 3.60	D	20.39 ± 7.24	D	55.91 ± 2.86	D
LS1B.3	33.42 ± 5.53	D	16.31 ± 4.02	D	46.51 ± 3.78	D
LS1B.4	38.03 ± 4.49	D	22.96 ± 7.06	D	49.76 ± 8.64	D
LS1C.1	35.38 ± 3.59	D	30.90 ± 3.18	D	50.17 ± 1.57	D
LS1C.5	38.80 ± 1.46	D	23.61 ± 4.83	D	55.15 ± 5.60	D
LS1D.2	50.85 ± 5.85	D	34.76 ± 2.90	D	45.20 ± 10.56	D
LS1D.3	44.02 ± 4.83	D	35.84 ± 4.38	D	44.51 ± 2.40	D
LS1D.3.2	50.09 ± 1.04	D	34.76 ± 6.76	D	55.77 ± 0.96	D
LS2A.1	32.39 ± 2.57	C	18.03 ± 10.95	E	52.32 ± 5.52	D
LS2A.2	41.71 ± 6.07	D	47.21 ± 1.70	D	51.21 ± 1.53	D
LS2A.4	43.42 ± 0.90	D	21.89 ± 14.02	D	55.70 ± 4.04	D
LS2A.5	55.47 ± 3.68	D	42.06 ± 3.22	D	60.75 ± 5.28	B
LS2B.1	51.45 ± 7.44	D	21.46 ± 6.14	D	61.44 ± 3.87	D
LS2B.3	57.78 ± 2.57	D	42.06 ± 9.91	D	55.70 ± 6.74	D
LS2C.3.2	43.85 ± 3.78	D	39.70 ± 8.74	D	58.47 ± 4.61	D
LSD2.4	36.50 ± 5.18	E	23.82 ± 10.19	D	56.81 ± 6.07	B
LSD2.5	40.43 ± 5.97	D	15.67 ± 8.94	D	59.30 ± 0.78	D

**Table 2:** Antagonistic activity of twenty *Usnea* ELF against the three plant fungal pathogens. The type of fungal interaction for each isolate against each pathogen and the percentage of growth inhibition (I%) ± standard deviation (SD) was indicated.

Isolate Codes	*C. gloeosporioides*		*C. cladosporioides*		*F. oxysporum*	
	Percent Inhibition (I%) ± SD	Type of Interaction	Percent Inhibition (I%) ± SD	Type of Interaction	Percent Inhibition (I%) ± SD	Type of Interaction
BG1A.5	23.01 ± 2.11	E	43.79 ± 3.01	E	39.00 ± 2.11	D
BG1B.1.1. A	25.61 ± 4.68	E	38.32 ± 7.63	E	27.66 ± 1.28	D
BG1B.1.1. B	30.73 ± 9.85	B	36.76 ± 1.57	D	28.45 ± 3.71	D
BG1B.1.2	45.56 ± 10.53	E	36.08 ± 2.36	C	33.14 ± 1.83	E
BG1B.2.2	39.15 ± 1.54	B	38.47 ± 1.57	D	34.90 ± 2.46	D
DBG1B.3.2	18.80 ± 1.84	E	29.05 ± 0.51	E	25.32 ± 1.06	D
BG1B.4.1	42.36 ± 7.30	D	36.08 ± 3.35	E	40.08 ± 10.08	D
BG1C.5.1	41.55 ± 1.20	E	26.99 ± 5.28	E	17.01 ± 0.83	D
BG1D.1	38.65 ± 0.17	E	24.59 ± 1.36	E	33.82 ± 4.69	D
BG1D.5	45.56 ± 3.84	D	34.36 ± 1.07	E	30.50 ± 0.21	E
BG2B.4	30.73 ± 4.26	E	23.39 ± 5.20	E	18.18 ± 0.21	D
BG2C.3	26.62 ± 1.84	E	36.59 ± 6.20	E	26.54 ± 2.07	D
BG3A.1.1	38.95 ± 0.76	E	42.25 ± 0.89	E	30.65 ± 0.83	D
BG3A.5	22.61 ± 4.52	E	29.73 ± 1.57	E	13.20 ± 13.38	E
BG3C.4	43.66 ± 2.08	D	53.73 ± 1.19	D	27.27 ± 3.01	D
BG4B.5.1	24.61 ± 5.09	D	36.42 ± 4.73	E	33.53 ± 9.37	D
BG4C.1	41.05 ± 11.84	E	34.70 ± 4.31	E	16.03 ± 4.99	E
BG5A.4.2	32.73 ± 3.59	E	32.39 ± 1.65	E	28.74 ± 3.00	D
BG5D.2	31.23 ± 5.04	D	38.99 ± 5.45	D	32.75 ± 3.98	D
BG5D.4	42.76 ± 1.88	E	46.36 ± 0.79	D	43.30 ± 0.29	D

## Data Availability

The original contributions presented in this study are included in the article/Supplementary Material. Further inquiries can be directed at the corresponding author.

## Supplementary Materials

The following supporting information can be downloaded at: https://www.mdpi.com/article/10.3390/jof11040302/s1, Figure S1: Antagonistic assay of the twenty *Ramalina* ELF isolates against *C. gleosporoides*; Figure S2: Antagonistic assay of the twenty *Ramalina* ELF isolates against *C. cladosporioides*; Figure S3: Antagonistic assay of the twenty *Ramalina* ELF isolates against *F. oxysporum*; Figure S4: Antagonistic assay of the twenty *Usnea* ELF isolates against *C. gleosporoides*; Figure S5: Antagonistic assay of the twenty *Usnea* ELF isolates against *C. cladosporioides*; Figure S6: Antagonistic assay of the twenty *Usnea* ELF isolates against *F. oxysporum*; Table S1: Detailed colonial descriptions of the ELF isolates from *Ramalina* sp.; Table S2: Detailed colonial descriptions of the ELF isolates from *Usnea* sp.
