# Supplementary material for: Comparative Antagonistic Activities of Endolichenic Fungi Isolated from the Fruticose Lichens Ramalina and Usnea
Source: J Fungi (Basel). 2025 Apr 10;11(4):302. doi: 10.3390/jof11040302 (PMC12028183; doi:10.3390/jof11040302)
Supplement: Supplementary file 1 [file jof-11-00302-s001.zip › jof-3433427-supplementary.pdf]

Supplementary Table S1. Detailed colonial descriptions of the ELF isolates from *Ramalina* sp.

Isolates	Color and tint in colony	Hyphae	Colony surface texture	Elevation	Colony margin	Pattern	Pigment exuded	Average Growth (mm)/day ± SD
**LS1A.1**	Surface: white turning to cream as it matures Reverse: predominantly white	Minimal and semi-submerged	Semi-powdery (floury)	Raised in the center	Irregularly undulate and filamentous	Flowery	Clear to pale yellow exudates in the center	2.92 ± 0.03
**LS1A.3.2**	Surface: white turning to cream as it matures Reverse: predominantly white to off-white	Minimal and semi-submerged	Powdery (floury)	Flat	Irregularly undulate	Radial pattern	No noticeable pigments or exudates	1.98 ± 0.38
**LS1A.4**	Surface: white with dark glaucous green to black center as it matures Reverse: predominantly white with black center	Abundant aerial mycelia	Hair-like thin threads with denser cottony center	Slightly raised center	Spreading and filamentous	Zonate to radiate	Black pigmentation in the center	3.4 ± 0.34
**LS1B.1**	Surface: white with a slightly off-white or light gray center Reverse: predominantly white with black center	Moderate aerial mycelia concentrated in the middle part	Fluffy and cottony, especially in the center. Toward the edges, it becomes more diffuse and less dense.	Raised center	Uneven and shows a radiating filamentous pattern, giving it a slightly irregular edge.	Radial pattern has a dense, central core and radiates growth toward the periphery. The outer filaments spread and are	Clear to pale yellow exudates in the center	2.99 ± 0.15

						less dense compared to the center		
**LS1B.3**	Surface: white Reverse: off-white with greyish brown patches which connects as it matures	Minimal to moderate and semi-submerged	Powdery (floury)	Semi-embedded	Irregularly undulate	Flowery	Clear to pale yellow exudates near the center	2.21 ± 0.12
**LS1B.4**	Surface: white, with a denser white center Reverse: off-white	Moderate aerial mycelia	Hair-like thin threads with with a denser feather-like mycelia radiating from the center	Low convex; slightly raised in the center	Filamentous or fimbriate, with delicate, hair-like projections spreading outward	Radial with filamentous rays extending outward from the central core	No noticeable pigments or exudates	3.04 ± 0.29
**LS1C.1**	Surface: white to off-white Reverse: predominantly white with black center	Moderate aerial mycelia concentrated in the middle part	Fluffy and cottony, especially in the center. Toward the edges, it becomes more diffuse and less dense	Raised center	Uneven and shows a radiating filamentous pattern, giving it a slightly irregular edge	Radial pattern has a dense, central core and radiates growth toward the periphery. The outer filaments spread and are less dense compared to the center	Clear to pale yellow exudates in the center	2.86 ± 0.53
**LS1C.5**	Surface: white to off-white Reverse: predominantly white with brown center	Moderate aerial mycelia concentrated in the middle part	Fluffy and cottony, especially in the center. Toward the	Raised center	Uneven and shows a radiating filamentous pattern,	Radial pattern has a dense, central core, with irregular discontinuing	Clear to pale yellow exudates concentrated in	2.98 ± 0.01

			edges, it becomes more diffuse and less dense		giving it a slightly irregular edge	radiant line. The outer filaments spread and are less dense compared to the center	the middle part.	
**LS1D.2**	Surface: off-white to ash grey Reverse: predominantly white to ash gray	Moderately abundant, especially in the center	The center is powdery (floury), surrounded by zonation and radiating lines throughout the rest of the culture plate	Umbonate	Spreading and filamentous	Zonate with radiating lines	No noticeable pigments or exudates	2.66 ± 0.19
**LS1D.3**	Surface: white to ash grey Reverse: predominantly white with ash gray turning to black center	Abundant and firm aerial mycelia	Powdery (floury) to cottony with irregular zonation in the middle part	Umbonate	Spreading and filamentous	Radiate which can be visibly seen in the reverse side of the colony	No noticeable pigments or exudates	3.60 ± 0.42
**LS1D.3.2**	Surface: white with pale buff yellow center Reverse: predominantly white with ash gray	Moderately abundant	Cottony and fluffy	Semi-elevated	Irregularly lobate	Zonate and flowery	No noticeable pigments or exudates	2.16 ± 0.45
**LS2A.1**	Surface: white to ash grey with darker center Reverse:	Sparsely distributed mycelia	Cottony middle, with a dense central core. The	Umbonate	Filamentous or fimbriate with thread-like	Irregular with concentric growth rings	No noticeable pigments or exudates	2.32 ± 0.45

	predominantly white with black center		mycelia become diffuse and less dense as it moves outward		appearance at the edges			
**LS2A.2**	Surface: white, with a denser white center Reverse: off-white	Moderate aerial mycelia	Hair-like thin threads with with a denser feather-like mycelia radiating from the center	Slightly raised in the center	Filamentous or fimbriate, with delicate, hair-like projections spreading outward	Radial with filamentous rays extending outward from the central core A concentric ring is also evident, with pronounced radial spoke	Clear exudates concentrated in the center	3.31 ± 0.02
**LS2A.4**	Surface: white Reverse: off-white	Moderate aerial mycelia	Hair-like thin threads with with a denser feather-like mycelia radiating from the center	Slightly raised in the center	Filamentous or fimbriate, with delicate, hair-like projections spreading outward	Radial with filamentous rays extending outward from the central core A concentric ring is also evident, with pronounced radial spoke	No noticeable pigments or exudates	3.18 ± 0.14
**LS2A.5**	Surface: black central region with ash grey surrounding area and a uniformly white outermost part. Reverse: predominantly	Moderately abundant, especially in the center	Dense and fluffy in the center transitioning to fine and feathery at the edges	Semi-elevated	Irregular, filamentous to feathery-like	The central core is compact while the surrounding areas have a feather-like, radiating appearance	No distinct colored pigments other than shades of gray and white	3.66 ± 0.14

	white with black center							
**LS2B.1**	Surface: center has pale mouse-grey to greyish green color. The outermost areas are uniformly white. Reverse: predominantly white with olivaceous buff center	Abundant aerial hyphae, particularly in the peripheral regions where active growth is occurring	Center appears dense while the peripheral regions are fluffy to cotton-like structure toward the edges	The central area appears low to moderately elevated**.** The outer regions are more elevated.	Diffuse and irregular, with a slightly feathered appearance	Concentric growth pattern with a dense central core that radiates outward. The outer region has almost cloud-like or cottony appearance.	No noticeable pigments or exudates	2.75 ± 0.03
**LS2B.3**	Surface: White with faint shades of gray toward the center. Reverse: off-white	Moderate to high quantity of aerial hyphae	The central region appears smooth but somewhat granular. The outer area has a fluffy, cotton-like texture	Slight dome shape, with the elevation increasing gradually from the center toward the edges	Irregular and diffuse	Generally circular, with evenly distributed growth across the surface	Predominantly white, with little visible clear pigment other than the dark central area.	3.48 ± 0.07
**LS2C.3.2**	Surface: white to ash grey Reverse: predominantly white with ash gray turning to black center	Abundant and firm aerial mycelia	Powdery (floury) to cottony with irregular zonation in the middle part	Umbonate	Spreading and filamentous	Radiate which can be visibly seen in the reverse side of the colony	No noticeable pigments or exudates	3.24 ± 0.15
**LS2D.4**	Surface: white Reverse: off-white	Moderate aerial mycelia	Hair-like thin threads with with a denser feather-like	Slightly raised in the center	Filamentous or fimbriate, with delicate,	Radial growth pattern with more concentrated	No noticeable pigments or exudates	2.71± 0.34

			mycelia radiating from the center		hair-like projections spreading outward	growth at the center		
**LS2D.5**	Surface: white to ash grey Reverse: off-white	Moderate aerial mycelia	Cottony or velvety	Slightly raised at the center	Even and smooth, slightly filamentous	Radial, with a dense center and spreading outward in a circular pattern.	No noticeable pigments or exudates	2.86 ± 0.17

Supplementary Table S2. Detailed colonial description of the ELF isolates from *Usnea* sp.

Isolates	Color and tint in colony	Hyphae	Colony surface texture	Elevation	Colony margin	Pattern	Pigment exuded	Average Growth (mm)/day ± SD
**BG1A.5**	Surface: white to ash gray, with a very faint buff middle part. Reverse: off-white	Moderate aerial mycelia	Cottony or fluffy	Slightly elevated, particularly at the center	Irregular and undulate	Roughly circular and radial pattern	No noticeable pigments or exudates	3.06 ± 0.10
**BG1B.1.1. A**	Surface: white to ash gray. Reverse: predominantly white with ash gray turning to black center	Abundant and firm aerial mycelia	Powdery (floury) to cottony with irregular zonation in the middle part	Umbonate	Spreading and filamentous	Radiate which can be visibly seen in the reverse side of the colony	No noticeable pigments or exudates	2.85 ± 0.03
**BG1B.1.1. B**	Surface: white with a black or dark gray center. Reverse: off-white	Aerial mycelia is dense in the center	Cottony or wooly in the outer regions, while the center seems more compact	Slightly elevated, particularly at the center	Lobate, irregular, and diffuse	Center is denser with radiating lines	No noticeable pigments or exudates	2.98 ± 0.07
**BG1B.1.2**	Surface: mostly white with a darker central region with buff to buff-yellow center Reverse: white with lemon-chrome to orange -yellow	Minimal to moderate aerial mycelia	Cottony, with a distinctly fluffy texture. The center appears to be granular to powdery	Slightly elevated	Irregular undulate and feather-like	Radial, with a concentric differentiation between the darker spore-producing center and the lighter hyphal edges	Pale buff center, no visible exudates	1.61 ± 1.30

**BG1B.2.2**	Surface: central dark region (blackish-gray) surrounded by a dense white to off-white outer layer Reverse: predominantly white with darker center	Moderate to abundant aerial mycelia	Woolly or cottony at the center transitioning to fine thread-like mycelia	Slightly raised	Diffuse and feathery	Clear radial pattern, with concentric rings. The central dark region is surrounded by progressively lighter rings of hyphae, creating a layered look	No noticeable pigments or exudates	3.04 ± 0.25
**BG1B.3.2**	Surface: white to ash gray surrounding mycelia with cream center Reverse: predominantly white to ash gray with pale Sulphur-yellow middle part	Abundant hyphal growth	Woolly or cottony, with appearing like wrinkled edges	Umbonate	Irregular	Concentric ring pattern, with alternating bands of dense and less dense hyphae, giving it a ripple-like appearance	No noticeable pigments or exudates	2.28 ± 0.08
**BG1B.4.1**	Surface: white to ash gray Reverse: White to cream with pale mouse grey center	Moderate to abundant aerial mycelia in the center	Fluffy specially with the center, the edges cottony	Umbonate	Irregular and semi-lobate	Flowery	No noticeable pigments or exudates	3.61 ± 1.23
**BG1C.5**	Surface: white with a dense blackish or dark gray center surrounded by some yellow or cream-colored areas near the inner core.	Moderate to abundant aerial mycelia	Cottony, with a distinctly fluffy texture. The center appears to be granular to powdery	Slightly raised in the center	Irregular undulate and feather-like	Radial, with a concentric differentiation between the darker spore-producing center and the lighter hyphal edges	Pale buff coloration encircled in the center	2.53 ± 0.59
**BG1D.1**	Surface: white to ash gray with dark	Abundant aerial mycelia	Hair-like thin threads with	Slightly raised center	Spreading and filamentous	Zonate to radiate	The center has	2.44 ± 0.03

	glaucous green to center Reverse: white with black center		denser cottony center				blackish coloration	
**BG1D.5**	Surface: white to ash gray Reverse: white with cinnamon-buff center	Semi to moderate abundant	Cottony in the center, the surrounding mycelia appear to be more feather-like	Flat to semi-raised	Irregular, feather-like	Radiate from the center with zonation	No noticeable pigments or exudates	1.70 ± 0.58
**BG2B.4**	Surface: white to off-white Reverse: white with buff-yellow to lemon-cream radiating lines from the center	Moderately abundant	Cottony center with feathery to vein like surrounding mycelia	Semi-elevated center surrounding mycelia is flat	Irregular, feathery-like	Radiate	No noticeable pigments or exudates	2.40 ± 0.10
**BG2C.3**	Surface: white to off-white. Reverse: white with cream to buff-yellow center	Dense throughout the colony	Cottony or velvety, becoming woollier and more filamentous towards the periphery	Slightly raised in the center	Irregular and undulate	Central, circular growth pattern, with radiating hyphae that extend outward in a layered manner	No noticeable pigments or exudates	2.13 ± 0.43
**BG3A.1.1**	Surface: white to ash gray. Reverse: white	Moderately abundant	Cottony with less dense mycelia in the edges	Slightly raised in the center	Undulate	Roughly circular and radial pattern	No noticeable pigments or exudates	3.09 ± 0.53
**BG3A.**5	Surface: predominantly white to off-white Reverse: white	Minimal mycelia	Cottony center with reduced mycelia in the edges	Semi-submerged to flat	Irregular to semi-undulate	Flowery appearing to be layered	No noticeable pigments or exudates	2.70 ± 0.67

**BG3C.4**	Surface: white with cream to pale buff center Reverse: white	Moderately abundant, becomes sparser and more thread-like as they radiate outward	Powdery to granular center with feather-like and filamentous surrounding mycelia	Flat to Slightly elevated	Filamentous to feathery-like	Radiate with zonation	Pale yellow coloration in the center	1.96 ± 0.58
**BG4B.5.1**	Surface: White to ash grey Reverse: white	Abundant aerial hyphae, particularly in the peripheral regions where active growth is occurring	Center appears dense while the peripheral regions are fluffy to cotton-like structure toward the edges	Raised in the center reducing through the edges	Diffuse and irregular, with a slightly feathered appearance	Concentric growth pattern with a dense central core that radiates outward. The outer region has almost cloud-like or cottony appearance.	No noticeable pigments or exudates	4.60 ± 1.34
**BG4C.1**	Surface: White with black coloration radiating from the center Reverse: White with greyish brown turning black as it matures	Semi-submerged and moderately abundant	Minimal cottony center with feathery to vein like surrounding mycelia	Semi-flat	Irregular with feathery-like appearance	Radiate	No noticeable pigments or exudates	1.68 ± 0.36
**BG5A.4.2**	Surface: white with pale straw-yellow Reverse: white with cinnamon-buff center	Abundant in the center with reduced surrounding mycelia	The center is powdery (floury), surrounded by zonation and radiating lines throughout the rest of the culture plate	Umbonate	Spreading and filamentous	Zonate with radiating lines	Clear to pale yellow pigmentation in the center	3.41 ± 0.12

**BG5D.2**	Surface: center has pale mouse-grey to greyish green color. The outermost areas are uniformly white. Reverse: predominantly white	Abundantly aerial hyphae particularly in the peripheral regions where active growth is occurring	Center appears dense while the peripheral regions are fluffy to cottony	The central area appears low to moderately elevated	Diffuse and irregular	Concentric growth pattern with a dense central core that radiates outward. The outer region has almost cloud-like or cottony appearance	No noticeable pigments or exudates	1.68 ± 0.73
**BG5D.4**	Surface: ash gray with cream to pale buff center Reverse: white	Moderate aerial mycelia concentrated in the center	Fluffy and cottony, especially in the central region. Toward the edges, it becomes more diffuse and less dense.	Raised center	Irregular, filamentous	Radial pattern, with a dense, central core and radiating growth toward the periphery.	Clear to pale yellow pigmentati on in the center	2.25 ± 0.96

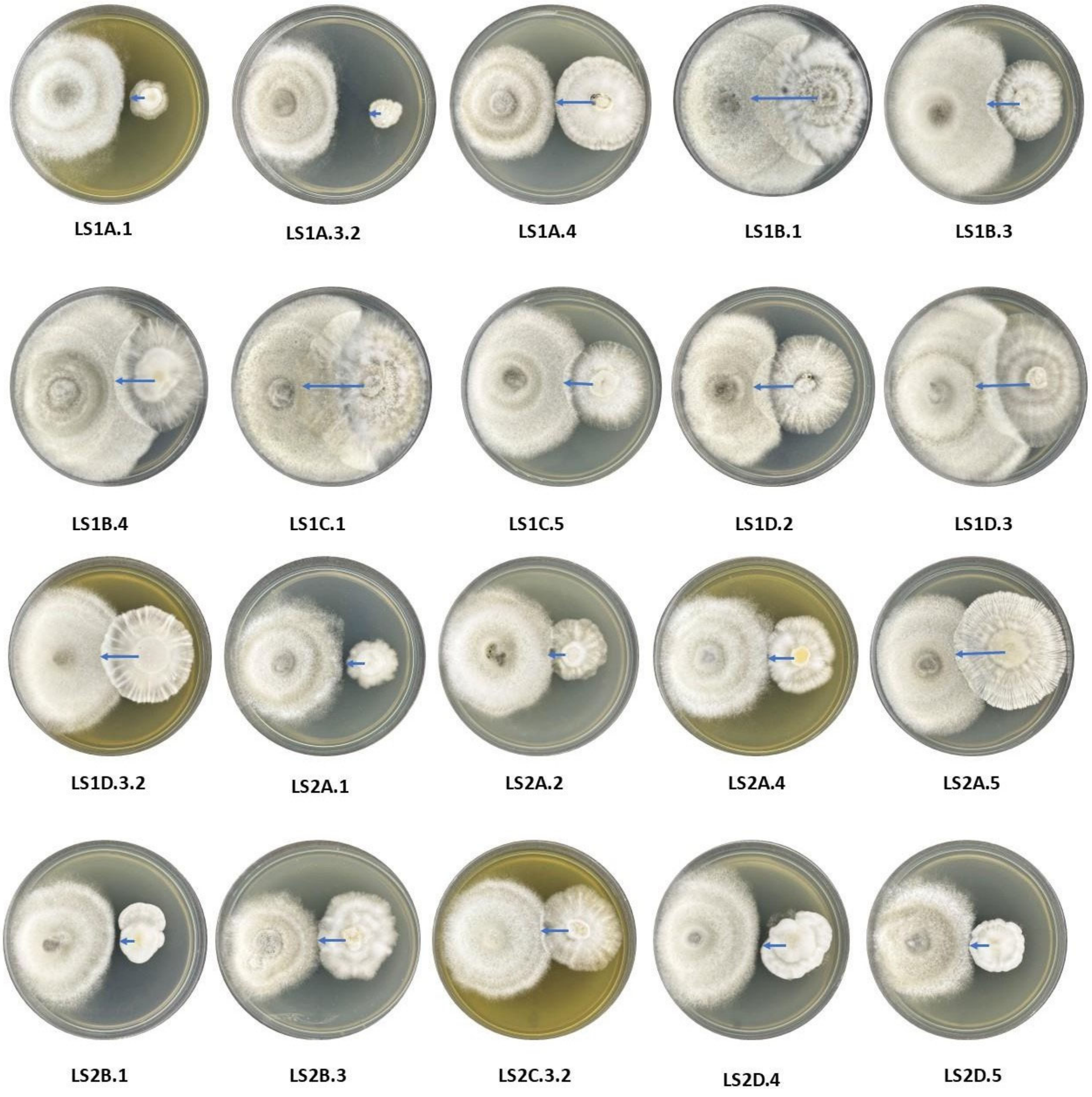

Supplementary Figure S1. Antagonistic assay of the twenty *Ramalina* ELF isolates against *C. gleosporoides*. The photograph was taken on the 10th day of incubation at PDA incubated at room temperature and ambient light.

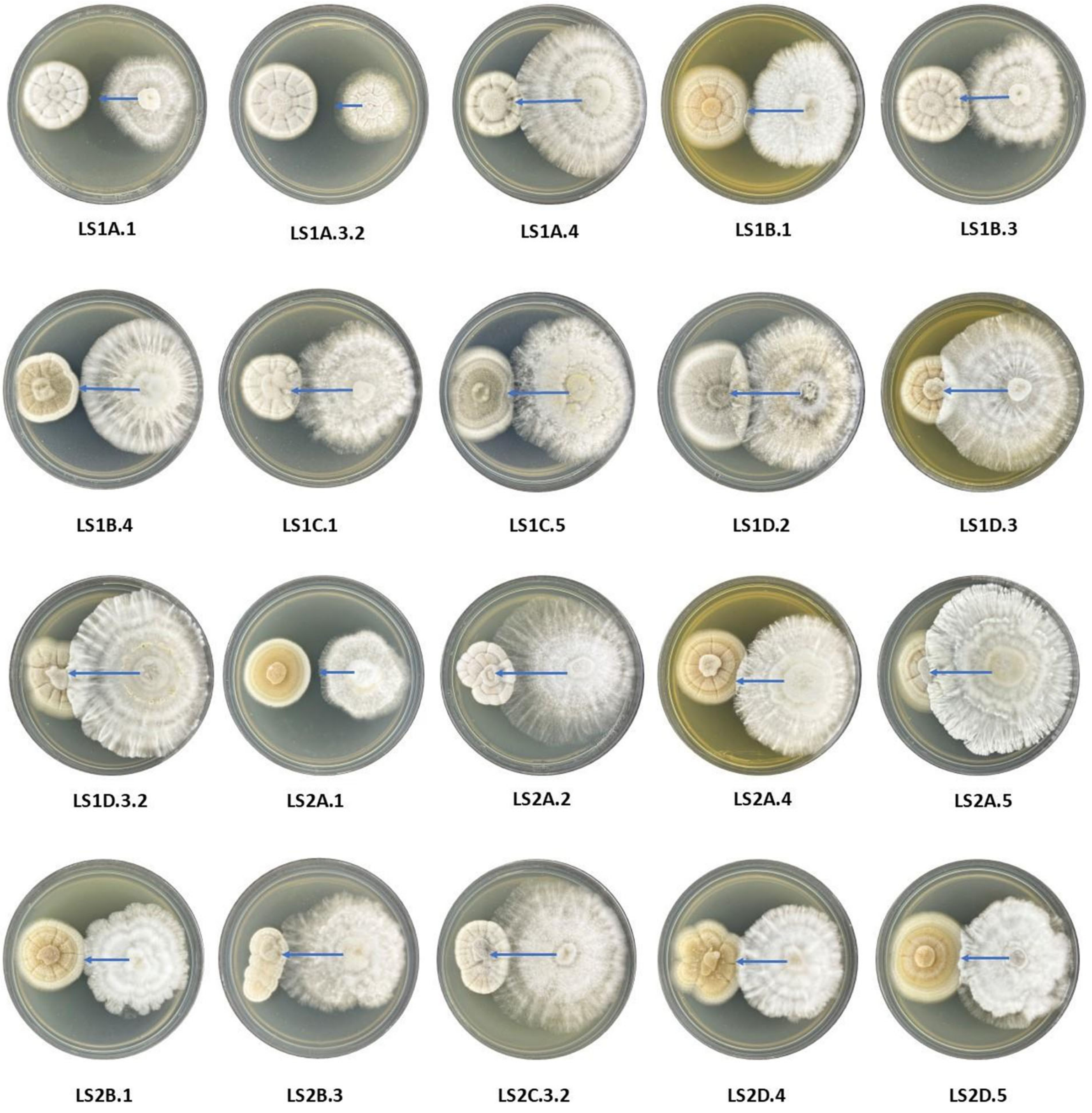

Supplementary Figure S2. Antagonistic assay of the twenty *Ramalina* ELF isolates against *C. cladosporioides*. The photograph was taken on the 10th day of incubation at PDA in room temperature and ambient light.

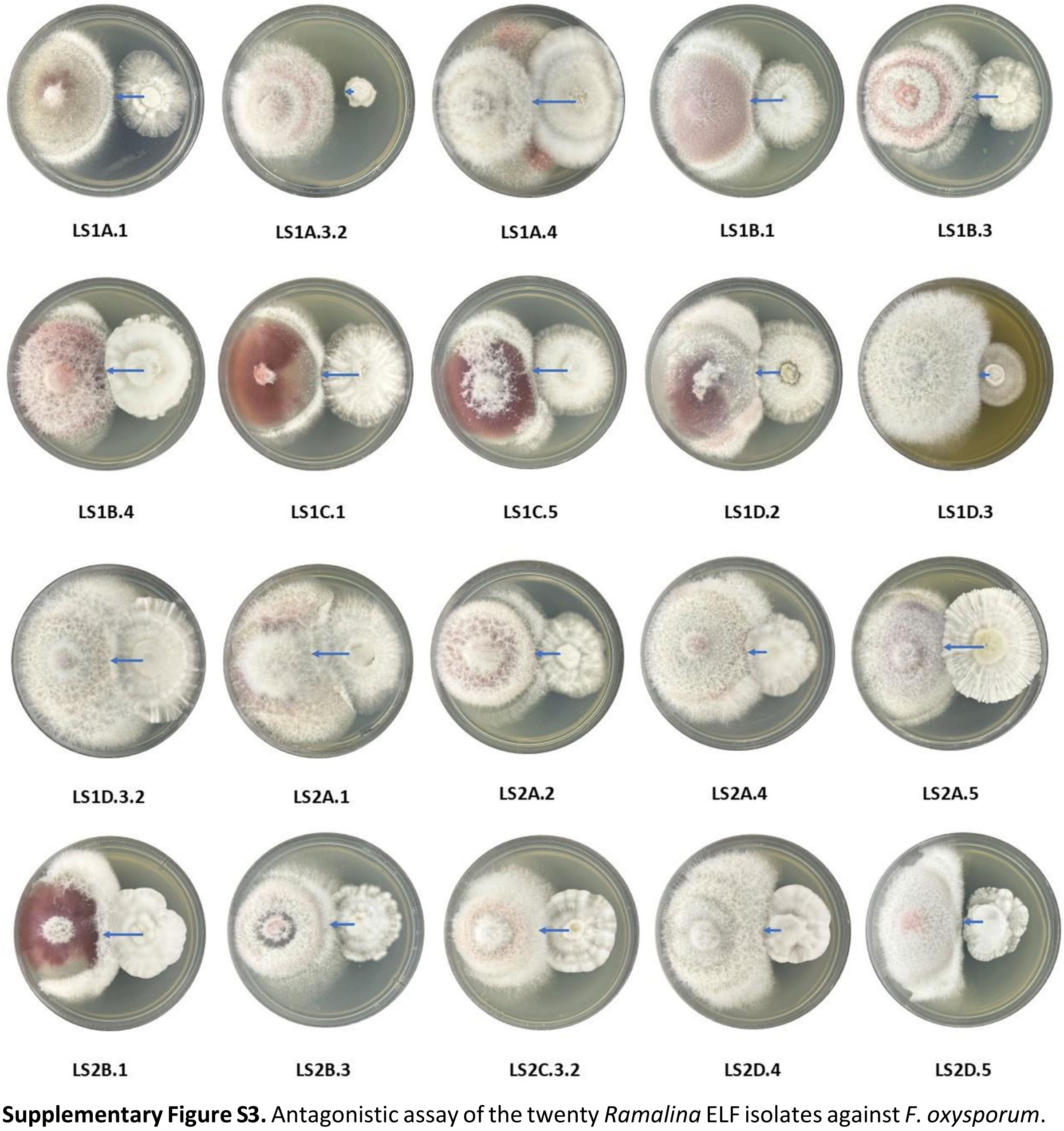

Supplementary Figure S3. Antagonistic assay of the twenty *Ramalina* ELF isolates against *F. oxysporum*. The photograph was taken on the 10th day of incubation at PDA in room temperature and ambient light.

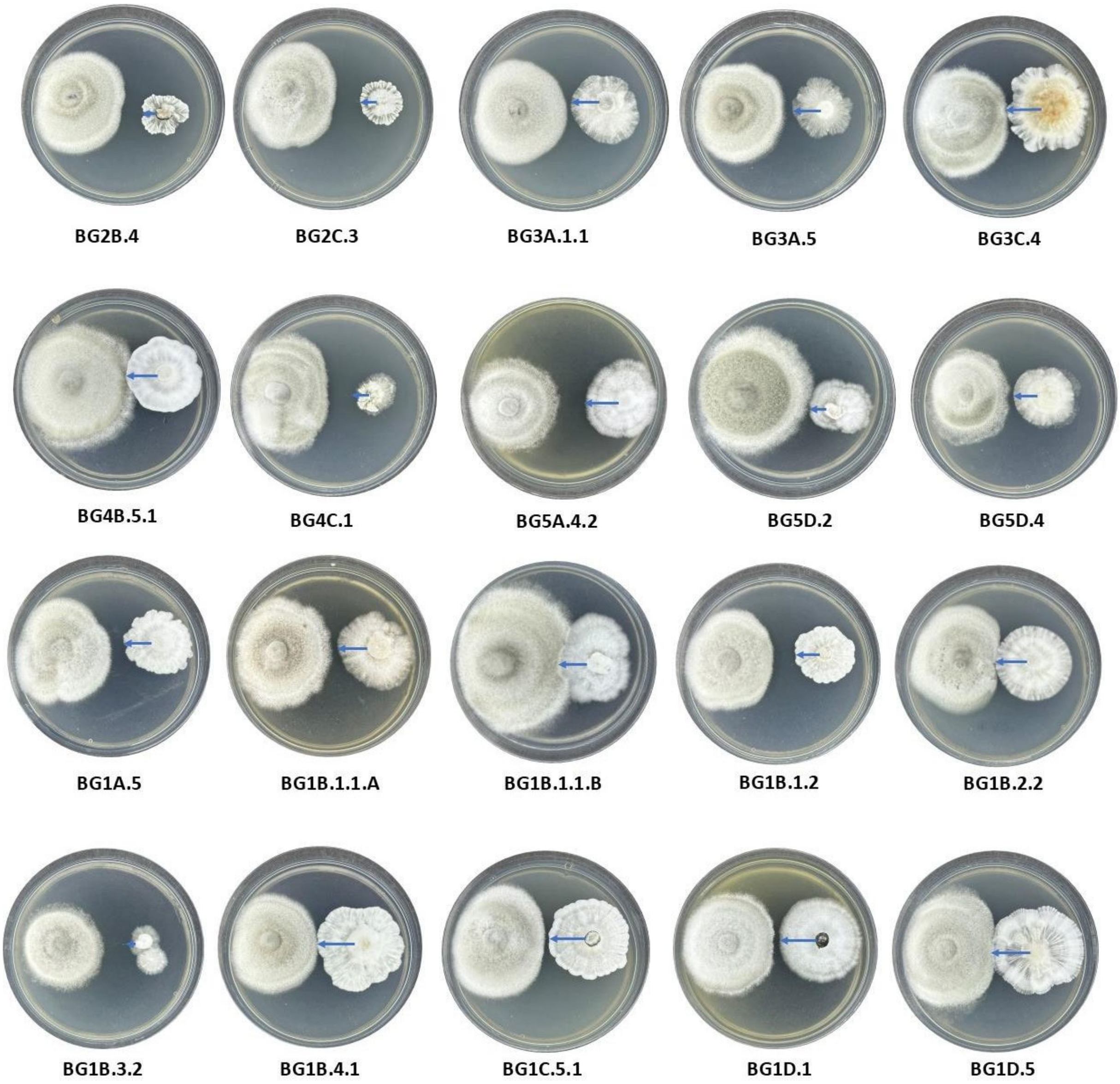

Supplementary Figure S4. Antagonistic assay of the twenty *Usnea* ELF isolates against *C. gleosporoides*. The photograph was taken on the 10th day of incubation at PDA in room temperature and ambient light.

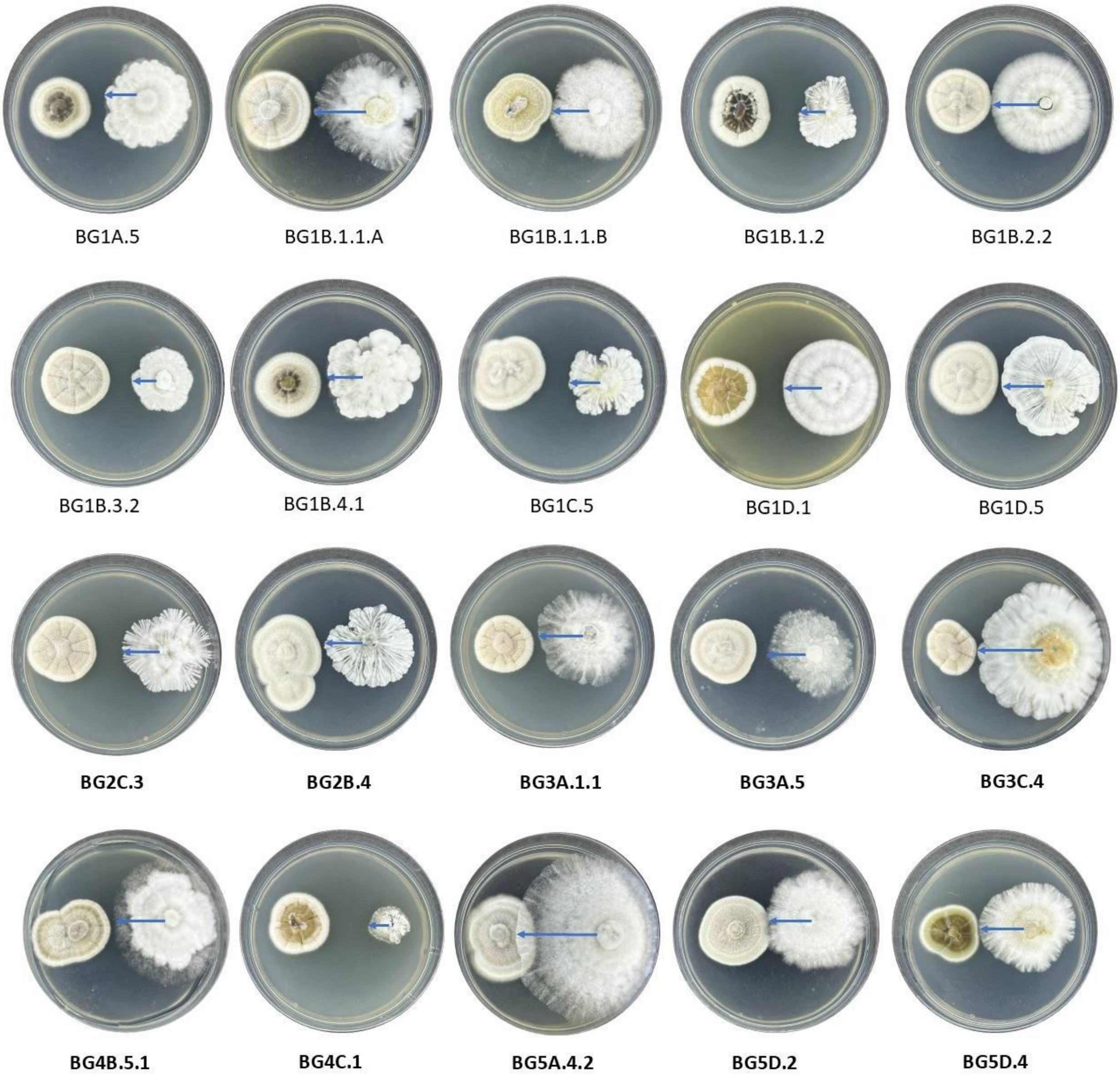

Supplementary Figure S5. Antagonistic assay of the twenty *Usnea* ELF isolates against *C. cladosporioides*. The photograph was taken on the 10th day of incubation at PDA in room temperature and ambient light.

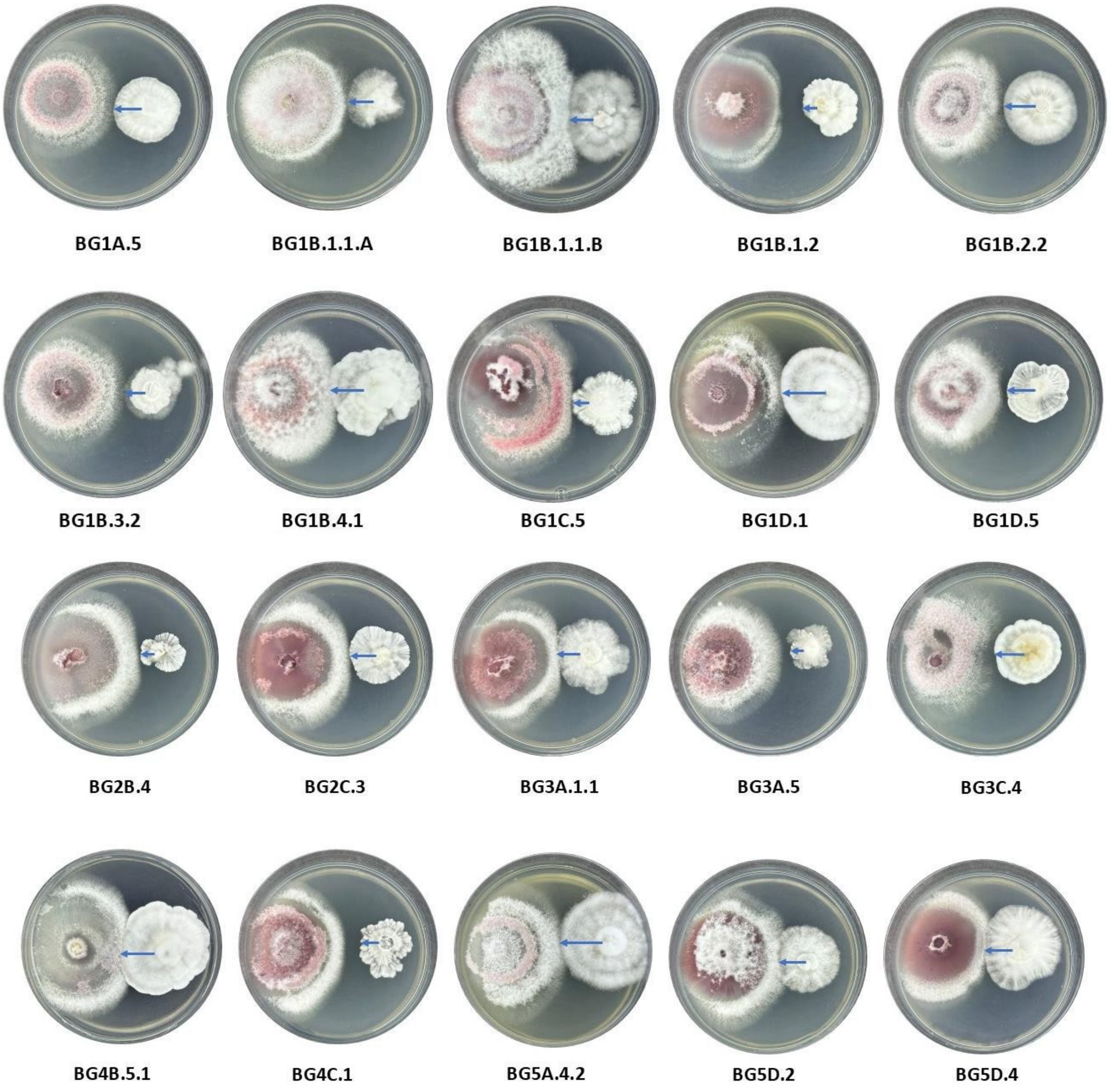

Supplementary Figure S6. Antagonistic assay of the twenty *Usnea* ELF isolates against *F. oxysporum*. The photograph was taken on the 10th day of incubation at PDA in room temperature and ambient light
